# Anatomy and physiology of the thick-tufted layer 5 pyramidal neuron

**DOI:** 10.3389/fncel.2015.00233

**Published:** 2015-06-26

**Authors:** Srikanth Ramaswamy, Henry Markram

**Affiliations:** Blue Brain Project, Ecole Polytechnique Fédérale de Lausanne, Campus BiotechGeneva, Switzerland

**Keywords:** pyramidal neuron, neocortex, dendrites, axon, synaptic transmission, back-propagating action potential, spike-timing dependent plasticity

## Abstract

The thick-tufted layer 5 (TTL5) pyramidal neuron is one of the most extensively studied neuron types in the mammalian neocortex and has become a benchmark for understanding information processing in excitatory neurons. By virtue of having the widest local axonal and dendritic arborization, the TTL5 neuron encompasses various local neocortical neurons and thereby defines the dimensions of neocortical microcircuitry. The TTL5 neuron integrates input across all neocortical layers and is the principal output pathway funneling information flow to subcortical structures. Several studies over the past decades have investigated the anatomy, physiology, synaptology, and pathophysiology of the TTL5 neuron. This review summarizes key discoveries and identifies potential avenues of research to facilitate an integrated and unifying understanding on the role of a central neuron in the neocortex.

## Introduction

The mammalian neocortex is endowed with a daunting diversity of neurons. These neurons are classified into excitatory pyramidal neurons and local circuit interneurons, which are predominantly inhibitory. Pyramidal neurons exist in all layers of the neocortex except layer 1, forming the most abundant neuron type. In particular, thick-tufted layer 5 (TTL5) neurons are one of the most extensively studied cell types in the neocortex (Larkman, 1991a; DeFelipe and Fariñas, 1992; Markram et al., 1997a; for review see Spruston, 2008).

**Figure 1:**
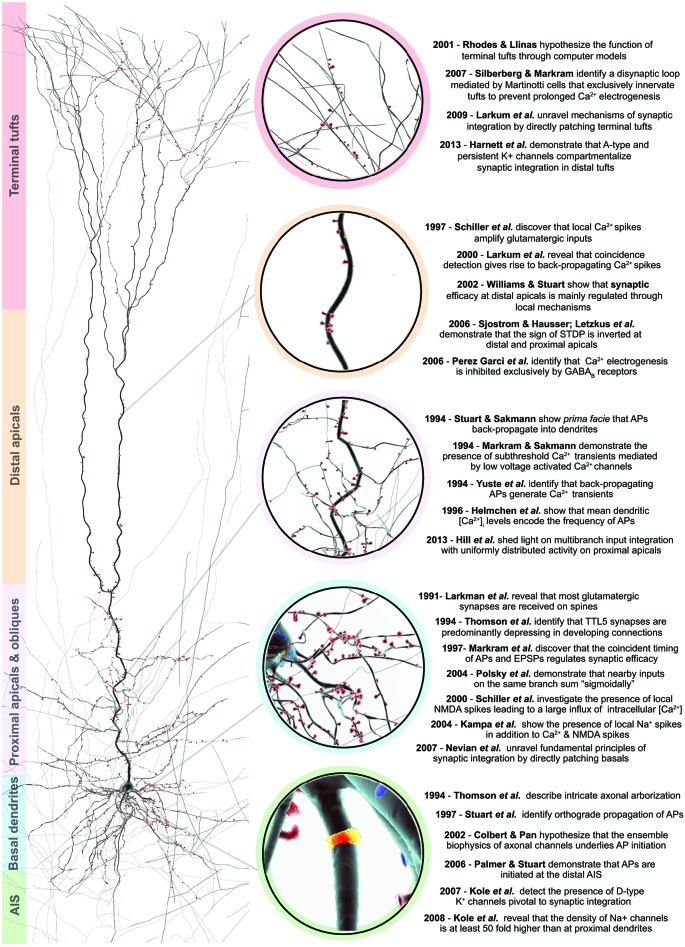
**An overview of salient discoveries in TTL5 structure and function**. Right, a 3D reconstruction of a biocytin filled thick-tufted layer 5 pyramidal cell. Spines are color-coded in red and depict the afferent excitatory synapses along the dendrites. The color bars to the left indicate the various morphological domains, which are ascribed to specialized functions. Center, the colored circles zoom-in to the morphological domain of interest. Left, a chronological summary of key structural and functional discoveries pertaining to the different morphological domains.

TTL5 neurons are predominantly found in lower layer 5 (layer 5B) and are characterized by a pyramid-like soma, and a stereotypical dendritic morphology with a prominent apical dendrite. In contrast to the other significant population of pyramidal neurons located in upper layer 5 (layer 5A) with slender apical dendrites, which project across hemispheres to the corpus callosum, TTL5 neurons mainly project to subcortical regions. Over the past two decades, *in vitro* patch-clamp recordings from brain slices using IR-DIC microscopy (Stuart et al., 1993) *in vivo* whole cell recordings and targeted recordings in freely moving animals (Svoboda et al., 1997; Murayama et al., 2007), and biologically detailed *in silico* models (Hay et al., 2011; Ramaswamy et al., 2012; Reimann et al., 2013) have significantly advanced our understanding of the structure and function of TTL5 neurons. This review attempts to consolidate the knowledge obtained from a vast body of existing literature in order to drive a unifying view of the anatomy, physiology, synaptology, and pathophysiology of the TTL5 neuron (see Figure 1).

## Generic Anatomy and Development

The generic anatomy of TTL5 neurons in rodent somatosensory cortex is characterized by a triangular soma, two distinct dendritic domains emanating from the base and apex of the soma (basal and apical dendrites, respectively), and a single axon sending out several collateral branches that further bifurcate and ramify exuberantly within the neocortex. The apical dendrites further branch into oblique dendrites and terminate with a crown-like thick tuft of dendrites in layer 1. There is a lack of quantitative data comparing the time-scales of anatomical development of TTL5 neurons across different cortical regions and species. Most of our knowledge on the development of TTL5 neurons can be attributed to studies in the frontal, parietal, temporal, and occipital lobes, in particular the visual, auditory, and somatosensory areas.

In sensory cortical areas, the anatomical development of TTL5 neurons is marked by three phases of growth; a fast growth phase of basal, oblique and apical branches from postnatal days 7 (P7) to 14 (P14), an intermediate localized growth phase of basal, apical and oblique branches growth from P14 onwards to postnatal day 21 (P21), and a slow growth phase from P21 to postnatal day 60 (P60) (Nicolai, 1981; Romand et al., 2011) We further direct the reader to exhaustive analytical studies on the overall anatomical development of TTL5 neurons (Nicolai, 1981; Kasper et al., 1994a; Zhu, 2000; Zhang, 2004) and rigorous quantifications of the morphometry of TTL5 dendrites (Larkman, 1991a,b).

### Somatic Development

The somata of TTL5 neurons have been subjectively classified according to triangular, round, and oval shapes (Romand et al., 2011). The three shapes are found to occur at all ages, however, somata appear to be mostly round or triangular between P7 and P14 and are predominantly triangular from P14 onwards. The somatic surface area, height and width increase markedly from P7 to P14. Although the growth of somata slows after P14, the height and surface area still increase significantly to reach the mature adult soma size at around P21 (Zhu, 2000).

### Dendritic Development

**Figure 2:**
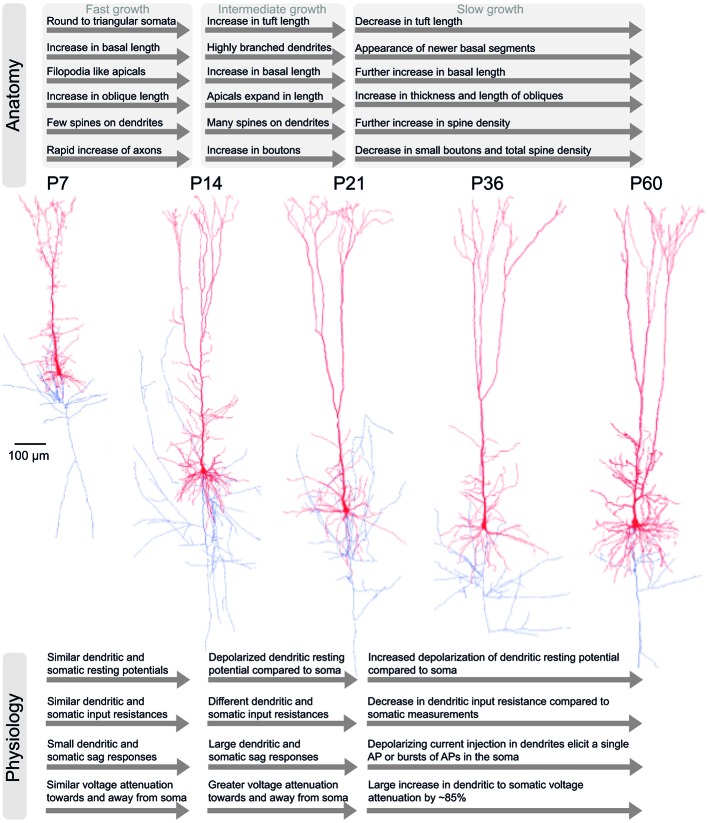
**Development of anatomical and physiological properties in TTL5 neurons**. An illustration of the salient anatomical (top) and physiological (bottom) properties in TTL5 neurons across different developmental time-scales (postnatal days (P) 7, 14, 21, 36, and 60). In the corresponding morphological reconstructions (middle), axons are shown in blue; soma and dendrites are shown in red.

Detailed morphological analyses have revealed that the TTL5 dendritic arbor increases in size and branching complexity during the first postnatal week (Wise et al., 1979; Zhu, 2000; Romand et al., 2011; see Figure 2 for a gantt-chart like illustration of the salient features of anatomical development). This period is also marked by the growth of secondary and tertiary branches of basal dendrites, the appearance of finer oblique dendrites, and an increase in the number of spines throughout the dendritic arbor. Between P7 and P14, basal dendrites also rapidly grow to increase their radial reach and path length. Concurrently, apical dendrites are typified by the appearance of several short and thin filopodia-like structures and oblique dendrites grow rapidly increasing both in length and thickness, reaching near maximal values. Furthermore, the tuft dendrites also increase significantly in length between P7 and P14. A wide diversity of tuft shapes, a signature feature of TTL5 neurons is already observed in this early growth period. By P7, spines are sparsely and homogeneously distributed throughout the basal dendrites. Between P7 and P14, spines located on the proximal dendrites are significantly reduced in number and continue to remain relatively sparse thereafter (Romand et al., 2011; Figure 2).

By P14, TTL5 neurons develop highly branched dendritic trees. From P14 until P21, basal dendrites continue to increase in radial reach and length in a trend consistent with earlier stages of growth, without the appearance of newer branches. The apical dendrites expand in length, reaching 92% of the maximal value observed in adult animals and the number of oblique dendrites and their lengths are established (Zhu, 2000; Romand et al., 2011; Figure 2). The previously observed filopodia-like structures on apical dendrites disappear almost completely during this period. While the intermediate branches of tuft dendrites do not show a significant increase in length, the terminal segments grow prominently between P14 and P21. This period of growth also undergoes a tremendous increase in spine density, attaining levels measured in adult animals (Larkman, 1991b; Figure 2).

During adulthood, the P21 to P60 growth period witnesses the emergence of newer basal dendritic segments with a further increase in length. The terminal segments of oblique dendrites increase in length and thickness. Contrastingly, the intermediate and terminal segments of dendritic tufts are shortened in length during this growth period (Romand et al., 2011; Figure 2).

In summary, three distinct developmental periods influence the dendritic growth of TTL5 neurons. The growth rate is fastest during the first period from P7 until P14, intermediate during the second period from P14 to P21, and the slowest during the third period from P21 to P60. In the first period basal, oblique, and tuft dendrites grow rapidly (Figure 2). Specific dendritic segments are both lengthened and thickened simultaneously, while tuft segments are only lengthened. During the second period, localized changes to specific compartments takes place and maximal spine densities. Thereafter during the third period, further localized changes continue at selected compartments and terminal segments. A characteristic feature of TTL5 dendritic development is that during a given period, segments are either lengthened or thickened, but never occurring concurrently at the same segment.

### Axonal Development

The TTL5 axonal arbor expands rapidly from P7 to P14, demonstrated through an increase in total length and segment number at these ages. At P7, the number of axonal collaterals is already equivalent to that of a fully mature TTL5 neuron (Romand et al., 2011; Figure 2). The radial distances from the soma to the local site of axonal collaterals increase significantly from P7 to P14, mirroring the growth of dendrites. A progressive lengthening of inter-segments between subsequent axonal stems is absent at from P7 to P14, but becomes prominent during P21 to P36 and higher ages.

The density of small boutons (diameters between 0.2 and 0.7 μm) increases between P7 and P14 while the density of large boutons (diameters between 1.0 and 1.8 μm) significantly increases between P14 and P21 (Figure 2). Adult levels of bouton density are attained by P21 (DeFelipe and Fariñas, 1992; Romand et al., 2011; Figure 2). As animals grow older, the density of small boutons tends to decrease along with the total bouton density (Romand et al., 2011; Figure 2).

## Occurrence Across Different Cortical Regions and Species

Previous studies have examined the occurrence of TTL5 neurons across different cortical regions and species (Jones et al., 1988; Rivara et al., 2003; Sherwood et al., 2003; Elston et al., 2011). These studies indicate that TTL5 neurons in different cortical regions and/or species are not merely scaled versions of a generic phenotype type, but can be structurally different while preserving fundamental stereotypical features (Elston and Jelinek, 2001; Elston, 2001; Jelinek and Elston, 2001).

The morphological complexity of TTL5 neurons progressively increases from primary sensory cortices located in the posterior occipital and temporal lobes, to primary and supplementary sensory-motor cortices situated in the parietal lobe, to the association and medial prefrontal cortices located in the frontal lobe.

In deeper layer 5B of primate visual cortex, Theodor Hermann Meynert first identified large solitary pyramidal neurons, which are distinguished by a single apical dendrite and numerous large basal dendrites. Meynert cells are known to occur in primary visual cortex of rodents, felines, higher order primates and humans (le Gros Clark, 1942; Chan-Palay et al., 1974; Peters and Kara, 1985; Akiyama et al., 1990; Anderson et al., 1998; for review see Roth and Dicke, 2005). The basal dendrites of Meynert cells bear more than 75% of spines received although they account for a much smaller proportion of the total dendritic length (Chan-Palay et al., 1974). The apical dendrite is progressively diminished of spines as it reaches the superficial layers and terminates without a prominent tuft in layer 1. Meynert cells in primary visual cortex share a number of morphological features in common with TTL5 neurons. However, Meynert cells differ characteristically in that their basal dendritic fields show elaborate branching and apical dendrites usually do not terminate with a thick tuft (Rockland, 1996; Thomson and Bannister, 2003).

Betz cells were first identified by Vladimir Alekseyevich Betz and are found in primary sensory-motor cortex of rodents, felines, higher order primates and humans (Betz, 1874; Walshe, 1942; Phillips, 1956; Kaiserman-Abramof and Peters, 1972; Rivara et al., 2003). Considered to be one of the largest neuronal types in the mammalian cerebral cortex, Betz cells are located in the medial sensory-motor cortex and account for about 10% of TTL5 neurons in deeper layer 5B (Rivara et al., 2003). Furthermore, the morphology of Betz cells is known to vary between the rostral and caudal parts of the primary sensory-motor cortex (Rivara et al., 2003). In human sensory-motor cortex, the total neuronal arbor volume of Betz cells is on average about 20 times higher than that of TTL5 neurons in motor cortex (Rivara et al., 2003; Sherwood et al., 2003). In comparison against TTL5 neurons, Betz cells contain several more proximal basal, apical and oblique dendritic segments, which profusely branch out from the soma (Braak and Braak, 1976).

In primate anterior cingulate and medial prefrontal cortices, the total expanse of the basal dendritic arbor of TTL5 neurons exceeds that of those in primary visual cortex by about five times (Zhang, 2004; Elston et al., 2005). This suggests that the morphological structure of pyramidal neurons is remarkably different between sensory and association cortices. In mature rodent medial prefrontal cortex, there are at least two distinct types of TTL5 neurons; simple pyramidal neurons, similar to pyramidal neurons found elsewhere in the neocortex typified by a single apical dendrite that terminates with a tuft in the superficial layers, and complex pyramidal neurons distinguished by early bifurcating dual apical dendrites and elaborately branching basal dendrites (Wang et al., 2006). Complex pyramidal neurons have almost twice the total apical dendritic length and significantly more extensive and frequently branching basal dendrites than the simple pyramidal neurons (Wang et al., 2006; van Aerde and Feldmeyer, 2015). In layer 5 of medial prefrontal cortex, the complex pyramidal neuron is the most commonly occurring form of pyramidal neuron, whereas the simple pyramidal neuron is more commonly found in other primary sensory cortices (Wang et al., 2006; van Aerde and Feldmeyer, 2015).

TTL5 neurons also display a remarkable morphological variability within different regions of the prefrontal cortex of higher order primates. A recent study has demonstrated that the total area of the basal dendritic arbor and spine density and can vary by more than 50% in the “average” pyramidal neuron across dorsolateral, medial and orbital prefrontal cortices (Elston et al., 2005).

## Projections to Cortical and Subcortical Targets

Long-range TTL5 axons target cortical and subcortical brain regions (Wang and McCormick, 1993; Kasper et al., 1994a; Brown and Hestrin, 2009). The elaborately branching TTL5 axon collaterals project across several neighboring neocortical microcircuits (Brown and Hestrin, 2009).

The TTL5 axon ramifies extensively in layer 5 with ascending collaterals projecting to the supragranular layers and descending collaterals projecting to infragranular layer 6 (Burkhalter, 1989; Keller, 1993; Thomson and Deuchars, 1994; Thomson and Bannister, 2003; Lübke and Feldmeyer, 2010). The supragranular layers receive targeted projections from a small number of TTL5 axon collaterals consisting of a small number of boutons (Thomson and Bannister, 2003). The innervation of pyramidal cells (PCs) in supragranular layers by ascending TTL5 axon collaterals is rather sparse, however, it is partially known that certain classes of GABAergic interneurons are targeted (Thomson and Morris, 2002).

Silver impregnation and retrograde labeling studies have indicated that the TTL5 axon projects to the superior colliculus via the cerebral peduncle, the pontine nuclei, tectal regions, the thalamic matrix, and to the striatum (Wise and Jones, 1976; Wang and McCormick, 1993; Kasper et al., 1994a; Morishima and Kawaguchi, 2006; for review see Thomson and Lamy, 2007). TTL5 axons exhibit a remarkable exactness in their projection pattern regardless of their origin from occipital, parietal or frontal regions.

TTL5 projections to different target areas are not only distinct in their anatomy but also in their physiology. A study combining neuroanatomical tract tracing with whole-cell recordings in coronal slices of adult rodent somatosensory cortex identified that TTL5 neurons projecting to thalamic and trigeminal nuclei were characterized with thick-tufts whose apical dendrites bifurcated with a wide branching pattern (Hattox and Nelson, 2007). The study by Hattox and Nelson unraveled a similarity in the physiological properties of TTL5 neurons depending on the projection area. Corticothalamic and corticotrigeminal TTL5 neurons shared similar intrinsic physiological properties, where a train of APs in response to depolarizing current injections exhibited an initial doublet, followed by insignificant or non-existent spike frequency accommodation (Hattox and Nelson, 2007). On the other hand, most TTL5 neurons projecting to the striatum were anatomically characterized by a narrow dendritic tuft, and displayed an initial AP singlet followed by pronounced spike frenquency adaptation of a train of APs (Hattox and Nelson, 2007).

## Dendritic Physiology

TTL5 neurons receive a bulk of glutamatergic synaptic inputs directly on basal, proximal apical and oblique dendrites (Thomson et al., 1993; Markram et al., 1997a) while GABAergic inputs are primarily received by the soma, proximal dendrites, the axon initial segment (AIS), and terminal tufts (for review see Somogyi et al., 1998). The physiological properties of TTL5 dendrites exert a profound influence in patterning synaptic input into action potential (AP) output through a highly complex transformation processes involving voltage gated ion channels, regenerative properties and integration gradients in single compartments (for reviews, see Johnston et al., 1996; Spruston, 2008; Branco and Häusser, 2010; Major et al., 2013). The physiology of TTL5 dendrites develops concurrently with anatomy.

### Development of Dendritic Physiology

The physiological properties of TTL5 dendrites mature in the first six postnatal weeks (Mason and Larkman, 1990; Kasper et al., 1994b; Zhu, 2000; Zhang, 2004). Between postnatal P2 to P14, the resting membrane potential and input resistance measured at the soma and dendritic tufts are very similar (Zhu, 2000; see Figure 2 for a Gantt-chart like illustration of salient features of physiological development). Depolarizing step current injections in both the soma and dendrite evoke brief all or none APs of comparable amplitudes at both locations, indicating the presence of a main regenerative Na^+^ conductance (Zhu, 2000). Hyperpolarizing step current injections elicit small sag responses at the dendrites and soma, indicating that dendrites already possess voltage gated hyperpolarization activated cation (I_h_) and muscarinic activated potassium (I_M_) conductances. The attenuation of dendritic responses measured at the soma (somatopetal) and somatic responses measured at dendrites (somatofugal) are similar, indicating that dendrites are electrotonically compact during this period (Zhu, 2000; Figure 2).

From P14 onwards to P28, the resting membrane potential and input resistance measured at dendritic tufts is significantly different from that at the soma. Suprathreshold depolarizing step current injections elicit APs at the soma. At P14, the duration of the dendritic regenerative potential outlasts the evoked somatic AP (Zhu, 2000; Figure 2). In response to hyperpolarizing current injections, the tuft and soma at P14 display pronounced sag responses (Zhu, 2000; Figure 2). Inward rectification, as shown by steady-state *I–V* relationships in the tuft indicates a selective increase in the dendritic I_h_ conductance (Zhu, 2000). Somatopetal and somatofugal voltage attenuation are almost 50% greater compared against the P2 period, showing that dendritic tufts increase in electrotonic distance from the soma.

In the next period between P28 to postnatal day 42 (P42), the resting potential at dendritic tufts is more depolarized than the soma. The dendritic input resistance is only slightly lower than at the soma. At P28, depolarizing step current injections at dendritic tufts are capable of eliciting either single APs or a burst of 2–3 APs at the soma (Zhu, 2000; Figure 2). The sag response to dendritic hyperpolarizing current injections appears to be more prominent at P28 as against P14 (Zhu, 2000; Figure 2). While depolarizing subthreshold dendritic current injections induce subthreshold reponses at the soma, prolonged depolarizing suprathreshold current injection results in a sustained depolarization plateau at dendritic tufts and causes bursts of APs at the soma (Zhu, 2000). The increased duration of dendritic regenerative potentials at P28 indicates an increased contribution of Ca^2+^ conductances, showing that during this period the ionic dependence of regenerative potentials switches from predominantly Na^+^ to Ca^2+^ (Zhu, 2000). Also, during this period, the dendritic tufts are remarkably isolated from the soma, causing a huge attenuation of dendritic voltage responses measured at the soma by a factor of almost 85% (Zhu, 2000; Figure 2).

Finally, between P42 and postnatal day 56 (P56), the dendritic and somatic membrane properties are similar to those measured in the previous period. The depolarizing sag response to hyperpolarizing dendritic current injection is very conspicuous and indicates further increased levels in the dendritic I_h_ conductance. Dendritic regenerative potentials evoked by depolarizing current injections give rise to bursts with 2–4 APs at the soma. During this period, the regenerative potentials at dendritic tufts attain their final kinetics, implying that Ca^2+^ conductances could have reached their peak densities (Zhu, 2000). The attenuation of dendritic voltage responses measured at the soma is comparable to the previous period of P28 to P42 (Zhu, 2000).

In summary, developing TTL5 neurons share many common properties with those of mature neurons after the second postnatal week (around P14). The apical dendrites are not fully mature until P42. The dendritic tufts switch from being electrotonically close to the soma at P2 to electrotonically distant at P14 (Figure 2). The ionic dependence of regenerative potentials in the dendritic tuft is changed from being Na^+^ dependent to Ca^2+^ dependent (Zhu, 2000). At P28, the regenerative dendritic potentials are still relatively small in amplitude and duration and often fail to elicit somatic APs (Zhu, 2000). In contrast, at P42 the dendritic regenerative potentials are often successful in evoking one or more somatic APs (Zhu, 2000).

### Voltage-Gated ion Channels in Dendrites

**Figure 3:**
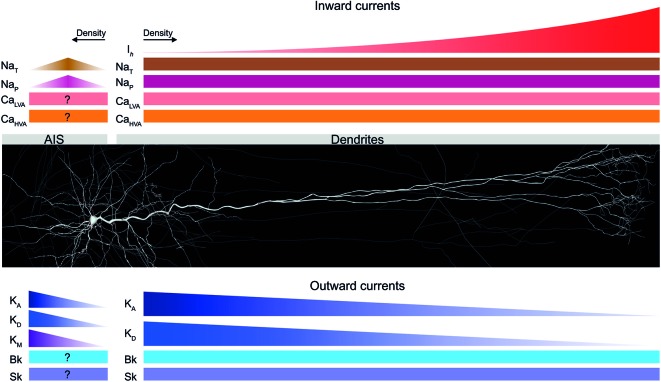
**Voltage-gated ion channels expressed in TTL5 dendrites and the axon initial segment (AIS)**. A schematic of the distribution profiles of different inward and outward currents expressed in the dendrites and AIS of TTL5 neurons. Inward currents are shown in shades of red; outward currents are shown in shades of blue. I_h_—hyperpolarization activated cation current; Na_T_—transient sodium current; Na_P_—persistent sodium current; Ca_LVA_—low voltage-activated calcium current; Ca_HVA_—high voltage- activated calcium current; K_A_—transient A-type potassium current; K_D_—delayed rectifier potassium current; K_M_—muscarinic-activated potassium current; Bk—big conductance calcium-activated potassium current; Sk—small conductance calcium-activated potassium current.

TTL5 dendrites express A-type and persistent K^+^ channels, transient and persistent Na^+^ channels, hyperpolarization-activated cation (HCN) channels, a plethora of Ca^2+^ channels, and small and large conductance (SK and BK, respectively) Ca^2+^ dependent K^+^ channels, all of which influence the integration of synaptic input (Stafstrom et al., 1985; Huguenard et al., 1989; Reuveni et al., 1993; Markram and Sakmann, 1994; Stuart and Sakmann, 1994; Crill, 1996; Kang et al., 1996a; Bekkers, 2000a,b; Korngreen and Sakmann, 2000; Reyes, 2001; Benhassine and Berger, 2005, 2009; Kole et al., 2006; Schaefer et al., 2007; Almog and Korngreen, 2009; Książek et al., 2013; Harnett et al., 2015; see Figure 3 for a schematic of the diversity and distribution profiles of voltage-gated ion channels in dendrites of TTL5 neurons).

A-type K^+^ channels decrease in density along the somato-dendritic axis and thus serve in defining a distal, low threshold region for the initiation of dendritic regenerative potentials (Bekkers, 2000a; Kang et al., 2000; Korngreen and Sakmann, 2000; Schaefer et al., 2007; Figure 3). A recent study has discovered that A-type and persistent K^+^ channels compartmentalize integration of input throughout the dendritic tuft (Harnett et al., 2013). In identifying new roles role for voltage-gated K+ channels in TTL5 dendritic tufts, the study by Harnett et al. (2013) proposes two important functional modes for K^+^ channels. *In vitro*, voltage-gated K^+^ channels act to regulate the interaction between the dendritic tuft, apical trunk, and axosomatic zones of information integration to exert influence on neuronal output. During sensory-motor behavioral tasks like active whisking, the high density of voltage-gated K^+^ channels in the apical dendritic arbor controls the threshold and time scale of regenerative plateau potentials. In addition, these intriguing findings also suggest that the high density of these channels perpetuate local plateau potentials in TTL5 distal dendritic compartments that are modulated by top-down excitatory inputs arriving at apical tufts (Harnett et al., 2013).

Transient Na^+^ channels occur with a uniform density along the somato-dendritic axis and are crucial in sustaining back-propagating action potentials (bAPs) and local dendritic spikes (Huguenard et al., 1989; Stuart and Sakmann, 1994; Figure 3). Persistent Na^+^ channels are also uniformly distributed along the somato-dendritic axis, and amplify the synaptic current in apical dendrites (Schwindt and Crill, 1995; Fleidervish and Gutnick, 1996; Mittmann et al., 1997; Astman et al., 2006; Figure 3).

HCN channels carry the depolarizing I_h_ current activated by hyperpolarization and are important for dendritic excitability. HCN channels increase in density by more than 50-fold from the soma to distal apical dendrites and shape the time course of synaptic input through spatially independent integration at apical and basal dendrites (Williams and Stuart, 2000; Berger et al., 2001, 2003; Lörincz et al., 2002; Kole et al., 2006; Harnett et al., 2015; Figure 3). Interplay between the I_h_ current and the membrane capacitance endows band-pass filtering abilities to TTL5 neurons, thus favoring dendritic resonance and frequency tuning (Ulrich, 2002). Recent evidence indicates that an age-dependent increase in dendritic HCN channel density ensures the development of TTL5 neurons from compact temporal integrators to compartmentalized integrators of basal and apical dendritic synaptic input (Atkinson and Williams, 2009).

A myriad of low (T-type) and high (L, N, P/Q and R types) voltage-gated Ca^2+^ channels also exist in TTL5 neurons (Reuveni et al., 1993; Markram and Sakmann, 1994; Almog and Korngreen, 2009). Ca^2+^ channels are critical in regulating neurotransmitter release, generating and sustaining regenerative dendritic events, and burst firing of APs (Schiller et al., 1997; Stuart et al., 1997a; Koester and Sakmann, 1998; Ohana and Sakmann, 1998; Williams and Stuart, 1999; Larkum et al., 2001; Figure 3).

Small and large conductance Ca^2+^ activated K^+^ channels (SK and BK, respectively) channels occur with a homogeneous density along the somato-dendritic axis of TTL5 neurons, and their activation reduces the occurrence of local dendritic Ca^2+^ spikes thus rendering dendrites less excitable. BK channels do not influence the temporal window to initiate back-propagating Ca^2+^ spikes, thus actively decoupling the axo-somatic and the dendritic AP initiation zones during high-frequency inputs (Kang et al., 1996a,b; Benhassine and Berger, 2005, 2009; Książek et al., 2013; Figure 3).

The cell-attached patch-clamp technique has long served as the method of choice to investigate the physiology of voltage-gated ion channels with the advantage of leaving the intracellular medium unperturbed. While this *modus operandi* has revolutionized our understanding of the properties of voltage-gated ion channels in central neurons, it is also prone to errors. A recent study by Williams and Wozny identified errors in the measurement of the density and kinetics of several voltage-gated ion channel types through cell-attached recordings from the soma and dendrites of central neurons in rodent brain slices (Williams and Wozny, 2011). Williams and Wozny demonstrated that such errors could be directly attributed to transmembrane voltages produced by the activation of voltage-gated channels attached to the tip of the recording pipette, and proposed remedial procedures to correct for associated errors (Williams and Wozny, 2011).

For further exhaustive information on the physiology of dendritic voltage gated ion channels in central neurons we guide the reader to three notable reviews (see Migliore and Shepherd, 2002; Trimmer and Rhodes, 2004; Lai and Jan, 2006).

### Dendritic Excitability and Local Regenerative Potentials

A culminating point in TTL5 dendritic function came about when Greg Stuart in Bert Sakmann’s laboratory performed direct patch-clamp recordings apical dendrites *in vitro* and demonstrated *prima facie* that single axo-somatic AP back-propagate into the dendritic arbor (Stuart and Sakmann, 1994). A bAP serves as a retrograde signal, conveying the level of neuronal output activity to the dendrites. Indeed, bAPs attenuate in amplitude along the somato-dendritic axis as they propagate from proximal to distal locations (Stuart and Sakmann, 1994; for reviews see Stuart et al., 1997b; Waters et al., 2005). Preliminary computational modeling studies have indicated that the morphology of the TTL5 dendritic arbor influences the back-propagation of APs (Vetter et al., 2001).

Another discovery, also in Bert Sakmann’s laboratory, provided the first evidence that EPSPs caused a transient increase in [Ca^2+^]_i_ mediated by the opening of low voltage activated Ca^2+^ channels (Markram and Sakmann, 1994). This study imparted additional insights that dendrites serve to function more than mere “passive cables” during low frequency synaptic activity. Furthermore, bAPs in proximal apical dendrites were also found to evoke transient [Ca^2+^]_i_ (Yuste et al., 1994; Markram et al., 1995; Schiller et al., 1995; Helmchen et al., 1996). TTL5 distal apical dendrites amplify glutamatergic inputs through local Ca^2+^ spikes, thus controlling the synaptic efficacy of cortico-cortical inputs (Schiller et al., 1997).

TTL5 dendrites operate as coincidence detectors by summing local spikes from individual branches with other inputs to evoke AP firing. Conventionally, this implies the coincident activation of a sufficient number of inputs to reach AP firing threshold (for reviews see Segev and London, 2000; Yuste et al., 2000; London and Häusser, 2005). A “critical frequency” of AP firing (~100 Hz) can elicit single regenerative Ca^2+^ spikes in apical and basal dendrites (Larkum et al., 1999a; Kampa and Stuart, 2006). While local Ca^2+^ spikes are generated upon dendritic synaptic input coincident with bAPs, their initiation can be restrained by dendritic GABAB receptor mediated inhibition (Pérez-Garci et al., 2006). A hallmark discovery by Matthew Larkum showed that the coincident summation of distal synaptic input with a bAP led to a so-called back-propagation activated Ca^2+^ spike (BAC) causing a burst of APs at the soma as a direct consequence (Larkum et al., 1999b).

Previous studies inferring the integrative properties of thin TTL5 dendrites have either employed computational modeling or optical imaging techniques (Schiller et al., 2000; Antic, 2003; Milojkovic et al., 2004, 2005; Kampa and Stuart, 2006). Although informative, a drawback of optical imaging as an experimental technique is that it is largely qualitative and prone to ambiguity in interpreting results as opposed to quantitative characterization. In a set of ground-breaking experiments, Thomas Nevian, Matthew Larkum and Jackie Schiller pioneered direct dendritic patch-clamp recordings to reveal the elementary principles of synaptic integration in TTL5 basal dendrites (Nevian et al., 2007). Simultaneous dendritic and somatic recordings of spontaneous events in TTL5 basal dendrites revealed a dendrite to soma EPSP attenuation of up to 40 fold at locations as far as 140 μm away from the soma. Contrastingly, the backward spread of EPSPs from the soma to basal dendrites experienced very little attenuation. Björn Kampa and Greg Stuart demonstrated that bAPs invading the TTL5 basal dendritic arbor depolarized the distal basal dendrites during high-frequency burst firing following the generation of local dendritic Ca^2+^ spikes (Kampa and Stuart, 2006). Nevian et al. (2007) further provided critical insights on the attenuation of bAPs in TTL5 basal dendrites and reported that when scaled to the relative size of apical and basal dendrites the attenuation of bAPs in both arbors were almost identical.

In addition to local Ca^2+^ spikes, Na^+^ and NMDA spikes can also be initiated in the TTL5 dendritic arbor (Schiller et al., 2000; Kampa and Stuart, 2006; Rhodes, 2006; Nevian et al., 2007; Major et al., 2008; Larkum et al., 2009; Polsky et al., 2009). By means of glutamate uncaging, Jackie Schiller was the first to demonstrate that synaptically evoked potentials in basal dendrites were in fact NMDA spikes, usually followed by a large local Ca^2+^ influx (Schiller et al., 2000). This study also identified that localized NMDA spikes potentially confer a degree of parallel processing and independent decision-making in TTL5 basal dendritic branches.

### Dendritic Integration of Synaptic Input

A long-standing view has held that TTL5 dendrites integrate synaptic input either linearly or supra-linearly (Cash and Yuste, 1998; Nettleton and Spain, 2000; for review see Gulledge et al., 2005). Biophysical modeling approaches have suggested that the “arithmetic” of local input summation in thin dendrites endows them with an ability to serve as independent computational subunits, which sigmoidally modulate their inputs prior to global summation (for review see Silver, 2010). An elegant study by Alon Polsky and Jackie Schiller combined confocal imaging and dual-site focal synaptic stimulation of TTL5 basal dendrites to reveal that nearby inputs on the same dendritic branch summed sigmoidally, whereas widely separated inputs or inputs to different branches summed linearly (Polsky et al., 2004). These findings by Polsky et al. (2004) provided experimental support for previous theoretical postulates of a two-layer neural network model of thin-branch dendritic integration in pyramidal neurons (Poirazi et al., 2003; see also Häusser and Mel, 2003).

In an extension to the above findings, Hysell Oveido and Alex Reyes further discovered that when barrages of input were injected into TTL5 apical dendrites *in vitro* to simulate synaptic activity, summation of inputs at proximal dendritic locations and the soma were sublinearly proportional to the intensity of synaptic barrages (Oviedo and Reyes, 2012). On the other hand, the contrary was observed at more distal dendritic locations where inputs added supralinearly to begin with and transformed to a sublinear summation regime with higher input activity (Oviedo and Reyes, 2012). More recently, experimental advances have demonstrated the integration of synaptic input in single dendritic branchlets by selectively stimulating single spines (see Branco and Häusser, 2010). Landmark experiments by Tiago Branco at Michael Häusser’s laboratory brought to light the remarkable fact that single dendritic compartments are not only sensitive to the direction but also the velocity of incoming synaptic input (Branco et al., 2010). Unitary spines were progressively activated through controlled uncaging of glutamate centripetally from a single dendritic branchlet towards the soma and centrifugally away from the soma towards a selected dendritic compartment. The centripetal activation always gave rise to somatic EPSPs of larger amplitude as opposed to the centrifugal activation (Branco et al., 2010). The EPSP amplitude and AP output were unfailingly sensitive to the direction of activation and activation of NMDA receptors (Branco et al., 2010). The succession of experiments identifying modes of synaptic convergence have undoubtedly reinforced the fact that the different TTL5 dendritic domains are specially equipped to discriminate against the spatiotemporal sequence of afferent synaptic input. To facilitate synaptic integration, it appears that the multitude of active mechanisms in TTL5 dendrites have devised ingenious schemes of interplay that are highly dependent on the localization, strength, sequence, and distribution profiles of inputs.

Previous computational modeling studies have hypothesized plausible explanations for synaptic integration in terminal apical tufts (Rhodes and Llinás, 2001) yet these fine structures have proven rather evasive for direct experimental access. In another milestone for dendritic research, Matthew Larkum et al. went on to patch terminal tufts, revealing that NMDA spikes provide the basis for how distal synaptic inputs influence TTL5 neurons to reach AP firing threshold (Larkum et al., 2009). This paradigm shift not only provided the basis for parallel processing of top-down input received by terminal tufts but also led to a whole new unifying principle of TTL5 synaptic integration, where basal dendrites and terminal tufts integrate inputs through the recruitment of local NMDA spikes in AP initiation relative to the fixed apical Ca^2+^ and axo-somatic Na^+^ zones of integration.

How influential are TTL5 distal synaptic inputs in AP initiation against their proximal counterparts? Distal synapses are expected to exert a lesser degree of influence on axo-somatic AP initiation, mainly due to loss of charge following the flow of current from the dendrites to the soma and the axon (for review see Magee, 2000; Stuart and Spruston, 1998). This poses the following question: do distal synapses spatially scale their peak conductance to render themselves eligible to “veto” AP initiation (Häusser, 2001; Rumsey and Abbott, 2006; for reviews see Magee, 2000; Segev and London, 2000; Williams and Stuart, 2003a)? Contrary to a progressive somato-dendritic increase in synaptic conductance observed in hippocampal CA1 pyramidal neuron dendrites (Magee and Cook, 2000), Stephen Williams in Greg Stuart’s laboratory provided the foremost evidence that the amplitude and time course of both excitatory and inhibitory dendritic synaptic input in TTL5 neurons are influenced by voltage-gated conductances (Williams and Stuart, 2002, 2003b). As a counteracting design strategy, distal TTL5 dendrites exhibit site dependent mechanisms to regulate synaptic efficacy. Although distal TTL5 dendrites are ineffective sources of background somatic excitation, they play a powerful transient signaling role through coincidence detection (Williams and Stuart, 2002).

Thin distal dendrites give rise to large local synaptic responses reducing the driving force for synaptic current owing to their small diameters and high input impedance. Therefore, distal dendrites can considerably influence AP initiation by activating voltage-gated conductances, which increase charge entry and consequently initiate local dendritic spikes (for reviews see Magee, 2000; Spruston, 2008).

TTL5 dendrites are bombarded with synaptic input during active network states *in vivo* (Borg-Graham et al., 1998; Paré et al., 1998; Destexhe et al., 2001; for review see Destexhe et al., 2003). Computational modeling has predicted that such ongoing activity attenuates synaptic potentials as they propagate across the dendritic arbor (Bernander et al., 1991; Hô and Destexhe, 2000; for review see London and Segev, 2001; Chance et al., 2002; Rudolph and Destexhe, 2003). Ensuing synaptic activity therefore dictates the efficacy of input that activates dendritic spikes through local synaptic integration to forward-propagate to the axonal AP initiation site. This regime of distributed processing is believed to have implications on the computational power of cortical pyramidal neurons (Mel, 1993; Häusser and Mel, 2003; Williams and Stuart, 2003b). The subsequent question that arises is about the prevailing state of knowledge about information processing regimes under high-conductance states during active network states *in vivo*. The first experiments to test the existence of such a regime by mimicking *in vivo* like synaptic conductance levels *in vitro* were performed by Stephen Williams demonstrating that synaptic conductances are highly compartmentalized in TTL5 neurons, and that the dendritic arbor is optimized to independently carry out axo-somatic and apical dendritic integration under high conductance states (Williams, 2004). Additional evidence also suggests that distal excitatory synaptic inputs decisively control the synaptic output of TTL5 neurons, powerfully influencing neocortical network activity (Williams, 2005).

In summary, the goal of developing a model that unifies the input-output properties of TTL5 neurons appears to be within sight. In a recent review, Guy Major, Matthew Larkum and Jackie Schiller have proposed a road map towards a conceptual framework to identify fundamental abstractions marrying the principal input-output properties of TTL5 neurons (Major et al., 2013; see also Branco and Häusser, 2010). Furthermore, milestone *in vivo* experiments such as those by Daniel Hill et al. in Arthur Konnerth’s group are beginning to shed light on a plausible model of prevalent multi-branch input integration in TTL5 dendrites where synaptic input and dendritic activity are homogeneously spread throughout the basal and apical arbors (Hill et al., 2013).

### Function of Dendritic Spines

TTL5 dendrites are richly decorated with spines, which directly receive most of the excitatory synaptic input (Peters and Kaiserman-Abramof, 1970; Peters, 1987; Larkman, 1991b). Although the functional relevance of spines is not fully understood yet, computational modeling and *in vitro* slice experiments strongly support the notion that: (a) spines might serve to optimize the filling of a large number of afferent synapses onto a dendritic segment by increasing the available surface area (Stepanyants et al., 2002); (b) spines could check the diffusion of critical molecules away from the synaptic locus, serving as individual biochemical compartments (Koch and Zador, 1993; for review see Nimchinsky et al., 2002); (c) spines could play a regulatory role on the electrical properties of neurons (Koch and Zador, 1993; Yuste and Denk, 1995; Yuste et al., 2000; Tsay and Yuste, 2002; Araya et al., 2006, 2007, 2014; Palmer and Stuart, 2009; for review see Sala and Segal, 2014). The spine head is the site of a tiny, amorphous structure called the postsynaptic density (PSD). Prominent in excitatory synapses, the PSD houses AMPA and NMDA receptors (AMPAR and NMDAR, respectively) attached to large protein “signaling machines” that regulate the strength of synaptic transmission (Kennedy, 1997, 2000). *In vivo* imaging approaches have revealed the existence of spines with varying sizes in neocortical PCs, thin transient and thick persistent spines, suggesting functional roles in regulating time-scales for synaptic plasticity (Trachtenberg et al., 2002; Holtmaat et al., 2005, 2006).

## Axonal Physiology

The TTL5 AIS marks the origin of the axonal arbor and is exclusively innervated by GABAergic axo-axonic synapses established by Chandelier cells (Somogyi, 1977; Szabadics et al., 2006; for review see Somogyi et al., 1998). The primary collaterals of the TTL5 axon are unmyelinated and myelination progressively sets in at higher order axon branches (Palmer and Stuart, 2006; Shu et al., 2007). The principal functions of TTL5 axons involve the initiation and propagation of APs, excitation-release coupling, and integration.

Here, we summarize the physiology of the TTL5 axon and further direct the reader to comprehensive reviews on axon physiology in central neurons (see Debanne, 2004; Rasband, 2010; Debanne et al., 2011; Kole and Stuart, 2012).

### Initiation and Propagation of APs

Preliminary insights on AP initiation in the unmyelinated AIS were gained almost half a century ago (Coombs et al., 1957; Fatt, 1957; Fuortes et al., 1957; Eccles, 1964; Palay et al., 1968; Peters et al., 1968). Through the years, numerous studies have also suggested the initiation of local regenerative potentials within dendrites of neocortical pyramidal neurons (Amitai et al., 1993; Markram and Sakmann, 1994; Yuste et al., 1994; Schwindt and Crill, 1995; Schiller et al., 1997, 2000; Stuart et al., 1997b; Larkum et al., 1999b, 2001, 2009; Zhu, 2000; Polsky et al., 2004; Gordon et al., 2006; Kampa and Stuart, 2006; Nevian et al., 2007; Major et al., 2008). However, a growing body of evidence conclusively indicates that TTL5 APs are often initiated in the low threshold axon rather than at the site of dendritic synaptic input (Stuart et al., 1997a; Colbert and Pan, 2002; Palmer and Stuart, 2006; Kole et al., 2007b; Shu et al., 2007; Fleidervish et al., 2010). Lucy Palmer and Greg Stuart were foremost to demonstrate that AP initiation in TTL5 neurons occurs at the distal site of the AIS, about 35 μm away from the axon hillock (Palmer and Stuart, 2006).

How do APs propagate in TTL5 neurons following their initiation? APs in TTL5 neurons orthogradely propagate into the axonal arbor, and retrogradely propagate to invade the dendritic arbor (Stuart et al., 1997a). The retrograde propagation of APs signals the level of neuronal output to the dendritic tree (see Dendritic excitability and local regenerative potentials; (Amitai et al., 1993; Markram et al., 1995; Schiller et al., 1995; Stuart et al., 1997b; Larkum et al., 1999a,b, 2009; Kampa and Stuart, 2006; Nevian et al., 2007).

The occurrence of axonal APs have also been reported to precede somatic APs, with the latency difference between the onset of axonal and somatic APs increasing at distal axonal locations (Stuart et al., 1997a). The latency difference between the peak of somatic and axonal APs increase with axonal recordings more distal from the soma, leading to an AP conduction velocity estimate of about 300 μm/ms (Stuart et al., 1997a). Axonal APs back-propagating into the TTL5 dendritic tree undergo distance and frequency dependent attenuation (Stuart and Sakmann, 1994; Stuart et al., 1997a). The latency difference between the onset and peak of somatic and dendritic bAPs have also been found to increase as a function of distance from the soma (Stuart et al., 1997a).

Several unique features distinguish AP initiation in TTL5 neurons. One such prominent feature is the characteristic rapid rise at the foot of the somatic AP, which manifests as a “kink” (Naundorf et al., 2006; McCormick et al., 2007). Detailed axonal recordings and computational modeling have revealed that this kink in the AP is exclusive to TTL5 neurons (Shu et al., 2007) and could be attributed to axonal AP initiation, owing in part to the high density of Na^+^ channels housed in the AIS (Inda et al., 2006; McCormick et al., 2007). The structural evidence for a high Na^+^ channel density in the AIS of cortical pyramidal neurons is both plentiful and conclusive (Inda et al., 2006; Kole et al., 2008; Lörincz and Nusser, 2010). However, what functional relevance does this high density confer? Although it is tempting to subscribe to the interpretation that a high Na^+^ channel density renders a low threshold in the AIS to facilitate AP initiation, the dilemma remains far from resolved. Previous studies by Colbert and Pan suggested that the biophysics of axonal channels and not a high Na^+^ channel density underlies AP initiation in TTL5 neurons, whereas several recent studies have tried to affirm that AP initiation is aided by a high Na^+^ channel density in the AIS of TTL5 neurons (Colbert and Pan, 2002; Kole et al., 2008; Dulla and Huguenard, 2009; Hu et al., 2009). The density estimation of Na^+^ channels in the AIS has been rigorously pursued for the past several years and consistent interpretations have surfaced only recently. Independent studies by Kole et al. (2008), Hu et al. (2009), and Fleidervish et al. (2010) indicate that the density of Na^+^ channels in the TTL5 AIS is about 30–50 times greater than at the soma.

By combining direct axonal recordings and computational modeling, a recent study by Hallermann and colleagues showed that AP initiation in the AIS is potentially inefficient as it involves high metabolic costs necessary to restore and maintain Na^+^ and K^+^ gradients (Hallermann et al., 2012). In the same study, predictions derived from *in silico* models indicate that although the AIS and node of Ranvier together showed the highest metabolic cost per unit membrane area, APs back-propagating into the dendrites and forward-propagating into the AIS display the highest energy consumption levels in TTL5 neurons (Hallermann et al., 2012). Greater metabolic costs of AP initiation and propagation down the TTL5 axon are potential trade-off towards minimizing energy and maximizing the reliability and fidelity of high-frequency AP transmission (Hallermann et al., 2012).

### Voltage-Gated ion Channels in the TTL5 Axon

The TTL5 axon expresses an assortment of voltage-gated ion channels, which are essential for AP initiation and propagation, influencing the kinetics of APs, release of neurotransmitters and reliability of synaptic transmission (for review see Debanne et al., 2011). The myriad of Na^+^, Ca^2+^ and K^+^ conductances has been extensively studied in axon collaterals and terminals of central neurons (see Figure 3 for a schematic of the diversity and distribution profiles of voltage-gated ion channels characterized in the AIS of TTL5 neurons).

Voltage-gated Na^+^ channels (Na_v_) in the AIS of TTL5 neurons encode for the fast-inactivating transient (I_NaT_) and slow-inactivating persistent (I_NaP_) currents (Caldwell et al., 2000; Colbert and Pan, 2002; Astman et al., 2006; Hu et al., 2009). Recent evidence has conclusively demonstrated a higher density of functional Na^+^ channels in the AIS of TTL5 neurons as against the soma (Kole et al., 2008; Hu et al., 2009; Fleidervish et al., 2010; Figure 3).

Ca_v_2.1 (P/Q-type) and Ca_v_2.2 (N-type) channels have been recently discovered in the AIS of TTL5 neurons (Yu et al., 2010; Figure 3). These channels determine pyramidal cell excitability through activation of large conductance calcium-activated BK channels (Knaus et al., 1996).

The AIS of TTL5 neurons supports a high density of voltage-gated D-type K^+^ channels, which play a pivotal role in integrating slow sub-threshold input and sculpting the AP waveform and duration (Kole et al., 2007b; Foust et al., 2011; Figure 3). Furthermore, D-type K^+^ channels also regulate neurotransmitter release, critically modulate the efficacy of TTL5 synaptic connections, and determine the onset latency of post-synaptic responses at individual synaptic contacts (Bekkers and Delaney, 2001; Kole et al., 2007b; Boudkkazi et al., 2011; for reviews see Rasband, 2010; Debanne et al., 2011; Kole and Stuart, 2012).

Recent experiments have revealed that non-inactivating voltage-gated K^+^ channels modulated by muscarinic receptors in the AIS of TTL5 neurons play an important role in the initiation and conduction of APs (Battefeld et al., 2014). The muscarinic receptor modulated K^+^ current (I_M_), first observed in bullfrog sympathetic neurons (Brown and Adams, 1980), significantly influences neuronal excitability by virtue of being the only active current near AP threshold (Marrion, 1997). Battefeld and colleagues characterized I_M_ in the TTL5 AIS and found that this current is mediated by voltage-gated K_v_7.2/7.3 (KCNQ2/KCNQ3) heteromultimers (Battefeld et al., 2014). In addition, Battefeld et al. (2014) also discovered that K_v_7.2/7.3 co-clustered with Na_v_ in nodes of Ranvier. The co-clustering mechanism increased the availability of the transient Na_v_ current, and accelerated the upstroke of the AP as a result (Battefeld et al., 2014). Furthermore, staining and imaging experiments revealed that although K_v_7.2, K_v_7.3, and Na_v_ were all concentrated within the AIS, K_v_7 subunits were restricted to the distal portion of the AIS, whereas Na_v_ occurred throughout the AIS (Battefeld et al., 2014; Figure 3).

### Intrinsic Firing Properties

Voltage-gated axonal conductances contribute to AP initiation and influence the intrinsic properties of TTL5 neurons, such as the threshold for AP initiation, AP after-hyperpolarization and after-depolarization, and the firing mode. TTL5 neurons mostly respond to depolarizing somatic current injections through a distinctive firing pattern with spike-frequency adaptation, but can also discharge a burst of APs (Connors et al., 1982; McCormick et al., 1985; Chagnac-Amitai et al., 1990; Connors and Gutnick, 1990; Mason and Larkman, 1990; Silva et al., 1991; Amitai, 1994; Kasper et al., 1994b; Schwindt et al., 1997; Williams and Stuart, 1999; Schubert et al., 2001; Steriade, 2004; Groh et al., 2009).

Experiments and computational modeling have demonstrated that bursts of APs are generated through the activation of Ca^2+^ channels, prior to the back-propagation of APs into the TTL5 dendritic arbor (Rhodes and Gray, 1994; Mainen and Sejnowski, 1996; Williams and Stuart, 1999). The importance of burst firing is critically dependent upon the fidelity of information transfer (Lisman, 1997; Williams and Stuart, 1999). A pertinent question here is if all APs during a burst propagate reliably into the TTL5 axonal arbor resulting in neurotransmitter release, and then what are the postsynaptic changes following such release? It is unambiguous that postsynaptic responses between TTL5 neurons exhibit frequency-dependent depression during a low frequency train of APs less than 100 Hz (Thomson and West, 1993; Markram and Tsodyks, 1996).

The next question that arises is about the significance of burst firing in TTL5 neurons. Preliminary work by Stephen Williams and Greg Stuart revealed the significance of burst firing, indicating that bursts served to enhance synaptic coupling between TTL5 neurons through distinct and synergistic pre and postsynaptic amplification mechanisms during bursts of APs (Williams and Stuart, 1999). The activation of dendritic Ca^2+^ channels by bAPs was found to be crucial in the generation of burst firing in TTL5 neurons (Williams and Stuart, 1999). Recent studies have also shown that the distal axon, beyond the AIS influences the intrinsic excitability of TTL5 neurons (Kole, 2011). Na^+^ channels that generate a persistent current in the node of Ranvier facilitate the probability of burst firing, and maintain a critical role for the first node of Ranvier in the generation of high-frequency bursts in TTL5 axons (Kole, 2011).

## Ephaptic Coupling

In the central nervous system, ephaptic coupling broadly refers to either the coupling of adjacent axons arising due to ionic exchange between neurons or due to prevalent local electrical field potentials (Katz and Schmitt, 1940; Arvanitaki, 1942; Jefferys, 1995). Ephaptic coupling can influence AP initiation, synchronization and timing. Axon myelination is believed to inhibit ephaptic coupling.

Although widely studied in different brain regions (Taylor and Dudek, 1982; Draguhn et al., 1998; Bokil et al., 2001), the functional relevance of ephaptic coupling in TTL5 neurons has only been investigated recently (Anastassiou et al., 2011). Simultaneous somatic recordings from up to 12 TTL5 neurons revealed that extracellular electric fields generated ephaptically-mediated changes of less than 0.5 mV under subthreshold conditions. The small amplitude ephaptic changes resulted in APs, in particular for slow frequency fluctuations (<8 Hz) of the extracellular field (Anastassiou et al., 2011). Local field fluctuations due to ephaptic coupling in TTL5 neurons entrain both subthreshold and suprathreshold membrane potential changes to synchronize neuronal activity (Anastassiou et al., 2011).

## Synaptology

The anatomical and physiological properties of TTL5 synaptic transmission influence the dynamics of electrical activity within the neocortical microcircuit. *In vitro* paired recordings have provided fundamental insights on the anatomical and physiological properties of TTL5 synaptic connections. More recently, data-driven unifying *in silico* approaches to reconstruct the neocortical microcircuit have revealed the crucial roles of the intrinsic morphological diversity of TTL5 neurons in ensuring the robustness of synaptic physiology and anatomy in the local neocortical microcircuit (Ramaswamy et al., 2012).

### Anatomy of Synaptic Connections

Light and electron microscopic examinations of biocytin filled TTL5 neurons have revealed intricate details on the number and spatial innervation patterns of synaptic contacts (Markram, 1997; Markram et al., 1997a). TTL5 neurons are connected with a probability of ~10% through about 4–8 putative synaptic contacts (mean ± S.D. of 5.5 ± 1.1 contacts). However, a major caveat is that this numerical information is almost entirely based on *in vitro* recordings from 300–400 μm thick brain slices where axons and dendrites are potentially severed due to the slicing procedure, and could therefore be an underestimate. Synaptic contacts between TTL5 neurons are distributed throughout the dendritic arbor, however, despite the heterogeneity of spatial innervation, contacts are predisposed to occur at specific dendritic locations. A majority of potential synaptic contacts underlying connections between TTL5 neurons are formed on secondary and tertiary branches of basal dendrites, about 80–120 μm from the soma (see Figure 2). The densities of synapses on primary, secondary and tertiary branches of basal dendrites have been found to be strikingly similar (Markram, 1997). A given TTL5 neuron can also potentially innervate its own dendritic arbor, establishing a so-called autapse (Van Der Loos and Glaser, 1972; Lübke et al., 1996). Remarkably, the spatial locations of autapses in TTL5 neurons have been found to mirror that of synapses, implying common principles of synapse formation and organization in local TTL5 microcircuits (Lübke et al., 1996).

GABAergic interneurons innervate TTL5 neurons by establishing synaptic contacts preferentially onto proximal dendrites and soma, axon, distal dendrites and tufts (Somogyi et al., 1998; Markram et al., 2004; Thomson and Lamy, 2007). While basket cells mostly target proximal dendrites and somata of TTL5 neurons (Gupta et al., 2000; Wang et al., 2002), Chandelier cells exclusively target the AIS (Somogyi, 1977; Szabadics et al., 2006), and Martinotti cells innervate apical dendrites and tufts (Somogyi et al., 1998; Pérez-Garci et al., 2006; Silberberg and Markram, 2007).

From an estimated 300–500 TTL5 neurons in a local cortical module (diameter of 300 μm; Szentágothai, 1975), a connection probability of 10–15%, and about five potential synapses per connection, a single TTL5 neuron could be potentially innervated from as many as 40 neighboring TTL5 neurons, receiving about 200 afferent synapses (Peters, 1987; Song et al., 2005; Perin et al., 2011; Ramaswamy et al., 2012).

### Physiology of Synaptic Connections

Synaptic connections between TTL5 neurons in the juvenile rodent neocortex display characteristic short-term frequency dependent depression, with a high initial probability of neurotransmitter release (Thomson and West, 1993; Thomson et al., 1993; Thomson and Deuchars, 1997; Reyes et al., 1998; Reyes and Sakmann, 1999; Berger et al., 2009). Although this phenomenon is mostly ubiquitous across several neocortical areas in juvenile animals, a notable exception is the medial prefrontal cortex where the response of monosynaptic connections between TTL5 neurons is marked by facilitating synapses with pronounced augmentation (Wang et al., 2006; Berger et al., 2009). In the rodent prefrontal cortex, paired-pulse responses have demonstrated that the physiology of monosynaptic connections between TTL5 neurons is weakly depressing between P7 and P9 (Zhang, 2004). The synaptic responses thereafter transition from being weakly facilitating around P12, with a gradual increase of facilitation until P20 and beyond Zhang (2004). The switch from depressing to facilitating responses appears to occur around P9 (Zhang, 2004), although the underlying mechanisms are yet to be ascertained. Similar to an increase in the morphological complexity of TTL5 neurons from the occipital to the frontal areas at least as seen in juvenile rodents (see Occurrence across different cortical regions and species), a corollary which necessitates further experiments could be that synaptic physiology switches from being predominantly depressing in the sensory areas in favor of facilitating in the association areas. It has also been observed that monosynaptic connections between TTL5 neurons in the mature somatosensory cortex are predominantly facilitating (Atkinson and Williams, 2009). However, it is not yet known if the balance of TTL5 synaptic physiology tilts from depression to facilitation with a progression of age across all cortical areas and species.

A prominent trait of depressing TTL5 synaptic connections is that once a certain “limiting” activation frequency is surpassed, postsynaptic responses display a marked decrease in amplitude inversely proportional to the activation frequency, termed the “1/f rule of synaptic depression” (Abbott et al., 1997; Tsodyks and Markram, 1997). For instance, according to this rule the average amplitude of the postsynaptic response at an activation frequency of 40 Hz is half the amplitude as that at 20 Hz. The various mechanisms underlying frequency dependent synaptic depression in TTL5 neurons are not fully understood yet. However, the phenomenon of synaptic depression has been found to be independent of the activation of postsynaptic voltage-gated channels or polysynaptic dendritic inhibition or shunting (Markram and Tsodyks, 1996; for review see O’Donovan and Rinzel, 1997). Presynaptic factors could also influence depression. Some preliminary experiments with the bath application of 100 μM cyclothiazide to block AMPAR desensitization could not abolish depression altogether, however, the rate of depression was slowed and the rate of recovery from depression and the EPSP amplitudes were found to increase in response to a presynaptic stimulus with a train of APs (Markram, 1997). AMPAR desensitization could therefore help in sculpting TTL5 synaptic responses mediated by frequency-dependent depressing synapses (for reviews see Trussell and Fischbach, 1989; Jones and Westbrook, 1996).

Afferent GABAergic inputs onto TTL5 neurons display both frequency dependent depression and facilitation (Thomson et al., 1996; Gupta et al., 2000; Ali et al., 2007; Silberberg and Markram, 2007; for review see Thomson and Lamy, 2007). Previous studies have demonstrated that the very same axon of a TTL5 neuron innervating a neighboring TTL5 neuron through depressing synapses on one hand can also innervate a Martinotti-like GABAergic interneuron through facilitating synapses on the other (Markram et al., 1998). Alex Reyes in Bert Sakmann’s laboratory observed similar synaptic transmission principles in layer 2/3 PCs of the juvenile somatosensory cortex (Reyes et al., 1998). This implies a differential signaling mechanism in neocortical information processing regulated by selective synaptic modifications. While TTL5 connections onto Martinotti cells are marked by low release probability facilitating synapses, connections onto basket cells are mediated by high release probability depressing synapses (Wang et al., 2002). Most GABAergic interneurons types in the neocortical microcircuit are known to inhibit TTL5 neurons through high release probability depressing synapses. A notable exception to this homogeneity of GABAergic innervation is seen in connections from small basket cells to TTL5 neurons, where inhibition is mediated by low release probability facilitating synapses (Gupta et al., 2000). A recent study demonstrated a depolarizing effect of inhibition from Chandelier cells onto neocortical PCs by virtue of a depolarized GABAA reversal potential in the axon relative to perisomatic compartments (Szabadics et al., 2006). While this has not yet been observed directly in TTL5 neurons, it raises interesting questions on selective inhibition of the pyramidal AIS by Chandelier cells.

Inhibition from different interneuron types to TTL5 neurons are predominantly mediated by fast GABAA receptors. However, neurogliaform cells evoke slow, and long-lasting inhibition through a mélange of GABAA and GABAB receptors in TTL5 neurons (Tamás et al., 2003). Furthermore, it appears that distinct GABAergic microcircuits connect layer 1 interneurons via layer 2/3 interneurons to TTL5 neurons in rat neocortex (Jiang et al., 2013). Disynaptic circuits involving layer 1 neurogliaform cells, which formed reciprocal connections with groups of layer 2/3 interneurons, inhibited the distal apical dendrites and tufts of TTL5 neurons through a combination of GABAA and GABAB receptors. This disynaptic configuration powerfully inhibited dendritic spiking in TTL5 neurons through reciprocal inhibition of layer 1 neurogliaform cells and layer 2/3 interneurons combined with an electric coupling-mediated synchronizing mechanism (Jiang et al., 2013). On the other hand, single-bouquet cells in layer 1, which formed unidirectional inhibitory connections with several types of layer 2/3 interneurons, enhanced dendritic spiking in TTL5 neurons through a disynaptic disinhibitory configuration (Jiang et al., 2013). In general, TTL5 neurons receive strong inhibition form parvalbumin expressing neurons, moderate inhibition from somatostatin expressing neurons, and weak inhibition form vasoactive intestinal polypeptide expressing neurons (Pfeffer et al., 2013). Thus, an assortment of local interneuron types strategically modulates the global activity of TTL5 neurons.

The physiology of monosynaptic transmission between TTL5 neurons is extensively characterized through whole-cell recordings *in vitro*. Unitary EPSPs evoked by a single presynaptic AP in developing TTL5 synaptic connections displayed amplitudes ranging between 0.15–5.5 mV with a mean of 1.3 ± 1.1 mV, a mean EPSP onset latency of 1.7 ± 0.9 ms, a mean 20–80% rise time of 2.6 ± 2.3 ms, and a mean decay time constant of 40 ± 18 ms (Markram et al., 1997a). The rather wide range of EPSP amplitudes could potentially arise from the number of release sites, the probability of neurotransmitter release or the quantal size, all of which form the basis of the classical quantal model of synaptic transmission (Del Castillo and Katz, 1954; Korn and Faber, 1991). By means of statistical analysis, Alex Loebel et al. predicted that multiple release sites mediate synaptic transmission between TTL5 neurons (Loebel et al., 2009). However, further experimental corroborations are essential to ascertain this prediction. Synaptic transmission between TTL5 neurons is highly reliable with a low mean percentage of transmission failures of 14.3 ± 17.6, and a mean coefficient of variation (c.v.) of EPSP amplitude of 0.52 ± 0.37.

Unitary EPSPs in monosynaptic TTL5 connections are voltage dependent, with an increase in magnitude of the amplitude, decay time constant and the voltage time integral at membrane potentials higher than −60 mV (Markram et al., 1997a). The amplification of EPSPs at more depolarized membrane potentials could be attributed to several sources, including increased current flow through NMDA receptors (NMDAR), block of I_h_ currents, and activation of low-threshold Ca^2+^ or persistent somatic Na^+^ channels (Stuart and Sakmann, 1994; Schwindt and Crill, 1995). At hyperpolarized membrane potentials, the postsynaptic response between TTL5 neurons is mainly mediated by AMPA receptors (AMPAR) with fast kinetics, and at more depolarized membrane potentials NMDAR mediate the postsynaptic response with comparatively slow kinetics. Michael Haüsser and Arnd Roth devised a novel technique to quantitatively peel out the rise and decay time course of recorded AMPA conductances in TTL5 connections, which are estimated at about 0.2 ms and 1.7 ms, respectively (Häusser and Roth, 1997).

The time course of Mg^2+^ block and unblock of NMDAR determines the extent of their activation by depolarization and has critical implications for spike-timing dependent synaptic plasticity (STDP) by delivering precision to the temporal window (Kampa et al., 2004). A spatial concentration of receptor “hot-spots” along the TTL5 apical dendrite reveals a somato-dendritic gradient of glutamate sensitivity (Dodt et al., 1998). Interestingly, stimulation of these glutamate receptor hot spots facilitates the triggering of both Na^+^ and Ca^2+^ spikes, implying that these hot spots serve as initiation zones for dendritic regenerative potentials (Dodt et al., 1998; Frick et al., 2001). The repertoire of ionotropic glutamate receptors in TTL5 neurons also includes kainate receptors (KAR), although studies confirming their functional relevance are rather scarce. KARs display kinetics on time-scales similar to AMPARs and increase in density along somato-dendritic axis (Eder et al., 2003).

Prevalent polysynaptic pathways have been identified in neocortical layer 5, where an assortment of GABAergic interneurons mediates connections between neighboring PCs. It has also been discovered that PCs in supra-granular layers exert strong inhibitory effects on neighboring PCs through the direct activation of nerve terminals of GABAergic interneurons, bypassing their somato-dendritic domain (Ren et al., 2007). The dynamics of polysynaptic pathways through the mediation of GABAergic interneurons display a rich variety of temporal and spatial patterns, ensuring a critical balance of the yin of inhibition and yang of excitation for cortical function (for review see McBain and Fisahn, 2001). In a striking dichotomy, inhibition in polysynaptic pathways is induced by discharge of local PCs and excitation is caused by specific GABAergic interneurons (Kapfer et al., 2007; Silberberg and Markram, 2007; for review see Silberberg, 2008).

Silberberg et al. discovered that inhibitory responses were evoked in TTL5 neurons following presynaptic stimulation of individual neighboring TTL5 neurons with frequency dependent trains of APs (Silberberg and Markram, 2007). Strikingly, the probability for inhibition between TTL5 neurons was more than twice that of direct excitation and inhibitory responses increased as a function of rate and duration of presynaptic discharge. Simultaneous somatic and dendritic recordings conclusively attributed the locus of inhibition to distal dendrites. Whole-cell recordings from local TTL5 neurons and neighboring interneurons combined with morphological reconstructions confirmed that Martinotti cells were the GABAergic interneuron population mediating this interaction (Silberberg and Markram, 2007).

High-frequency discharge activates the Martinotti pathway rendering inhibitory interactions between TTL5 neurons, which are otherwise predominantly excitatory during low-frequency discharges. The Martinotti pathway prevents over-activation of TTL5 neurons, and is therefore crucial in preventing epileptoform activity (Pérez-Garci et al., 2006; Silberberg and Markram, 2007). By exclusively innervating distal tufts of TTL5 neurons in supra-granular neocortical layers, the Martinotti pathway serves a role akin to a fire-extinguisher by preventing the prolonged regeneration of dendritic Ca^2+^ spikes in TTL5 neurons and consequent high-frequency bursting, thereby maintaining cortical function by ensuring a balance of inhibition and excitation (Pérez-Garci et al., 2006). A recent study by Thomas Berger and colleagues revealed that the Martinotti pathway is not exclusive to the somatosensory cortex alone and is strikingly ubiquitous in its occurrence as a motif across a multitude of neocortical areas, however its precise role, for instance in synaptic plasticity entails further investigation (Berger et al., 2009).

Recent studies have demonstrated that the somatic depolarization of TTL5 neurons (>5 mV) substantially increases the amplitude and shortens the latency of the disynaptic inhibitory response in neighboring TTL5 neurons (Zhu et al., 2011). Paired recordings from TTL5 neurons have revealed that sufficient depolarization increases the EPSP amplitude, elevates the firing of interneurons and inhibition, thus reflecting the analog mode of synaptic transmission between TTL5 neurons and interneurons (Zhu et al., 2011). Therefore, it appears that membrane potential-dependent modulation of inhibition is a crucial strategy to dynamically balance neocortical yin and yang under different activity regimes.

### Properties of Afferent Synaptic Input and Modulation of TTL5 Function

The extensive arborization of the TTL5 dendrite facilitates the reception of synaptic connections from a medley of glutamatergic and GABAergic neurons. Descending axon collaterals from PCs originating in the lower confines of layer 2 and middle layer 3 establish synaptic contacts with oblique TTL5 dendrites (Thomson and Bannister, 1998; Reyes and Sakmann, 1999; Kampa et al., 2007). Although it is known that layer 4 spiny stellate cells form precise topographical projections to layer 5A, it is not completely clear whether deeper TTL5 dendrites are specifically innervated (Feldmeyer et al., 2005). The functional role of afferent inputs from layer 6 PCs on TTL5 dendrites is only superficially understood. One might speculate that the axon collaterals of cortico-thalamic PCs in layer 6 could target TTL5 dendrites in the upper confines of layer 5 during their ascending course towards the supragranular layers. A couple of recent studies suggest that the axons of cortico-cortical PCs in layer 6 are predisposed to form a plexus in layer 5, however, it is inconclusive if TTL5 dendrites are included in the target specificity of this plexus (Kumar and Ohana, 2008; Marx and Feldmeyer, 2012). Recent experiments *in vitro* have employed laser-scanning photostimulation techniques to focally uncage glutamate to characterize functional input from layer 6 excitatory neurons to TTL5 dendrites (Zarrinpar and Callaway, 2014).

**Figure 4:**
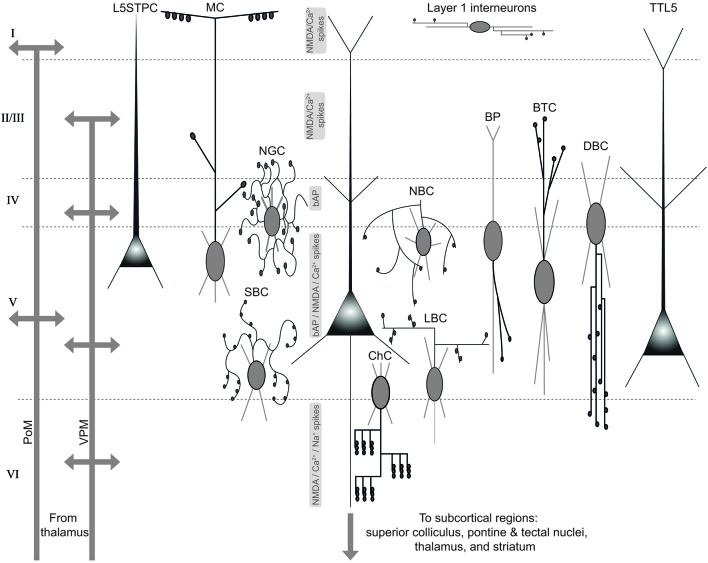
**Diverse sources of afferent excitatory and inhibitory input to, and targets of efferent excitatory output from TTL5 neurons**. An illustration of the TTL5 neuron in the local neocortical microcircuit showing diverse sources of excitatory and inhibitory inputs, and targets of excitatory output. Schematic representations of the various neuron types afferent to the TTL5 neuron are shown according to their known dendritic, peri-somatic or axonal innervation domains. Local electrogenic properties, and their likely layers of origin are shown to the left of the TTL5 schematic in the center. PoM—posterior medial nucleus; VPM—ventral posterior medial nucleus; L5STPC—layer 5 slender-tufted pyramidal cell; MC—Martinotti cell; NGC—neurogliaform cell; SBC—small basket cell; ChC—chandelier cell; LBC—large basket cell; NBC—nest basket cell; BP—bipolar cell; BTC—bitufted cell; DBC—double bouquet cell; TTL5—thick-tufted layer 5 pyramidal neuron.

The expansive TTL5 dendritic arbor can be demarcated into distinct functional domains based on characteristic afferent inputs received from GABAergic local circuit interneurons (see Figure 4 for a schematic of the diverse sources of afferent excitatory and inhibitory input to, and targets of efferent excitatory output from TTL5 neurons). The division of the TTL5 dendritic arbor into these functional domains endows enhanced capabilities for the integration of varied streams of synaptic input. Martinotti cells preferentially target distal TTL5 dendrites and terminal tufts, while small basket cells predominantly target the proximal apical and basal dendrites and the soma (Thomson et al., 1996; Kawaguchi and Kubota, 1997; Somogyi et al., 1998; Wang et al., 2002; Silberberg and Markram, 2007; Ali and Thomson, 2008). TTL5 innervation by nest and large basket cells appears to be rather heterogeneous, covering the entire dendritic arbor (Wang et al., 2002). Synaptic contacts from bipolar, double bouquet and bitufted cells are made onto the proximal and distal dendrites for the most part (for reviews see Somogyi et al., 1998; Markram et al., 2004; Figure 4). Chandelier cells exclusively target the TTL5 AIS through axo-axonic synaptic contacts (Somogyi et al., 1982; Howard et al., 2005; Szabadics et al., 2006). Neurogliaform cells in the supragranular layers innervate distal TTL5 dendrites through metabotropic GABAB receptors mediating slow inhibition (Tamás et al., 2003; Oláh et al., 2007; Figure 4). Furthermore, terminal TTL5 dendritic tufts are innervated by a local plexus of axons from GABAergic intereneurons residing in layer 1, also giving rise to slow inhibition through GABAB receptors (Zhu, 2000; Zhu and Zhu, 2004; Figure 4).

TTL5 dendrites directly receive thalamocortical afferents from the ventrobasal (VB) thalamus (Gottlieb and Keller, 1997; Figure 4). Furthermore, thalamic inputs involving lemniscal afferents arising from the ventroposterior medial nucleus (VPM) target proximal TTL5 dendrites (Ahissar et al., 2000; Feldmeyer et al., 2005; Lübke and Feldmeyer, 2007; Schubert et al., 2007; Figure 4). It is hypothesized that both slender and thick-tufted neurons in layer 5 function as integrators of paralemiscal and lemniscal thalamic afferents through weak but reliable monosynaptic connections arising from layer 4 spiny stellates (Feldmeyer et al., 2005). *In vivo* whole-cell recordings have revealed that thalamic afferents innervate neurons in layers 5B and 6, concurrently to those in layer 4 (Constantinople and Bruno, 2013; Figure 4). This study discovered that a multitude of thalamic neurons innervated pyramidal neurons in the deeper layers including TTL5 neurons, and identified a bistratified model of sensory information flow from thalamus to two parallel streams, comprising of an upper stratum of layers 4 and 2/3, and a lower stratum of layers 5/6 (Constantinople and Bruno, 2013; Figure 4). This bistratified model facilitated sensory information to fully circumvent upper layers. Synaptic afferents originating in thalamus reliably elicited APs in TTL5 neurons *in vivo*, implicating their roles in mediating behavior by virtue of their projection to other higher-order brain areas (Constantinople and Bruno, 2013).

Recent experiments have yielded a plethora of information on the modulation of the TTL5 neurons by both excitatory and inhibitory afferents. In particular, *in vivo* experiments have postulated critical roles of inhibitory interneurons in controlling TTL5 function in behaving animals (for a review see Palmer et al., 2012a). The advent of optogenetic approaches to selectively manipulate populations of neurons has now made it possible to study local electrogenic properties of TTL5 neurons including the initiation and sustenance of Na^+^, Ca^2+^, and NMDA spikes and their spatiotemporal synergies. In particular there is a better understanding of how at least two different forms of inhibition exclusively targeting distal dendrites and terminal tufts of TTL5 neurons modulate their function *in vivo* (see Figure 4 for a schematic of excitatory, and inhibitory afferents, and local electrogenic properties in the context modulation of TTL5 activity).

Martinotti cells receive facilitatory synaptic input from TTL5 neurons (Pérez-Garci et al., 2006; Kapfer et al., 2007; Silberberg and Markram, 2007). Burst firing in TTL5 neurons due to network activity inhibits Ca^2+^ spike generation in distal dendrites through disynaptic inhibition via the recruitment of Martinotti cells (Murayama et al., 2009; also see Palmer et al., 2012a; Figure 4). Although the exact function of this disynaptic circuit is yet to be ascertained, some studies indicate that disynaptic inhibition of TTL5 neurons could serve to synchronizing TTL5 network activity (Berger et al., 2010), while others suggest a modulation of the dynamic range of TTL5 neurons (Kapfer et al., 2007; Murayama et al., 2009; see also Palmer et al., 2012a). Dendritic Ca^2+^ spikes directly cause burst firing in TTL5 neurons (Schwindt and Crill, 1999; Williams and Stuart, 1999), therefore disynaptic inhibition could be an operating mechanism by which TTL5 neurons in a network that are recruited first shut out other TTL5 neurons from participating in network activity (see Palmer et al., 2012a). Disynaptic inhibition in TTL5 neurons *in vivo* mediated by Martinotti cells are brought about wholly by GABAA receptors operating on fast time scales (Murayama et al., 2009).

Neurogliaform cells in layer 1 directly inhibit the tufts of TTL5 neurons (Chu et al., 2003; Jiang et al., 2013; Lee et al., 2014; Muralidhar et al., 2014). By virtue of their morphology with a dense local axon, neurogliaform cells also inhibit other interneurons in layer 1 (Zhu and Zhu, 2004; Oláh et al., 2007). Neurogliaform cells potently inhibit TTL5 tufts through a combination of fast GABAA, and slow GABAB receptor activation (Tamás et al., 2003; Oláh et al., 2007, 2009; Figure 4). Recent *in vivo* experiments by Palmer and colleagues unraveled a cellular basis for interhemispheric inhibition of layer 5 pyramidal neurons through the activation of neurogliaform cells mediating inhibition through slow GABAB receptors (Palmer et al., 2012b). Elegant *in vivo* experiments by Palmer and colleagues revealed that GABAB mediated inhibition from neurogliaform cells exert a remarkable influence on the spiking activity of layer 5 pyramidal neurons, despite their confined and targeted innervation of distal dendrites (Palmer et al., 2012b). Although it is not very clear from the study if the implicated neurons were indeed TTL5 neurons with conspicuous thick-tufts terminating in layer 1, in general the local dendritic activity of layer 5 pyramidal neurons *in vivo* is durably suppressed through GABAB receptors by the activation of neurogliaform cells (Palmer et al., 2012b).

Thus, it appears that the function of TTL5 neurons *in vivo* is crucially governed by the selective activation of at least two distinct populations of GABAergic interneurons, which inhibit TTL5 activity across different time scales. Direct dendritic inhibition of TTL5 neurons through a combination of fast GABAA and slow GABAB receptors shuts off local electrogenic activity (Ca^2+^, and NMDA spikes) and modulates global TTL5 function *in vivo*. Further *in vivo* experiments are essential to peel out the roles of specific populations of GABAergic interneurons that selectively innervate targeted parts of TTL5 dendrites, soma and axons to better understand their normative circuit function in behaving animals.

## Synaptic Assemblies

Donald Hebb’s pioneering theoretical contributions to learning and memory have exerted a continuing influence on neuroscience and psychology for more than half a century. Hebb postulated that when one neuron persistently drives another in its neighboring vicinity, then the connection between them is strengthened (Hebb, 1949). Futhermore, Hebb proposed that as a consequence this can lead to the development of clustered synaptic coupling of neurons into assemblies of cells where experience sculpts their constituent network arrangement. Through the past several years, an accumulation of experimental evidence has corroborated Hebb’s postulates (see Dudai, 1989; Abeles, 1991; Kandel, 2007).

In spite of a vast body of experimental evidence for Hebb’s postulates, several previous studies have indicated that Hebb’s postulate of “neurons that fire together wire together” would lead to saturation of synapses within assemblies of cells, curbing their dynamic range and contracting memory storage capacity (Rochester et al., 1956; Rosenblatt, 1962; Bienenstock et al., 1982; McNaughton and Morris, 1987; Dayan and Abbott, 2001). Experimental studies have demonstrated that saturated long-term potentiation (LTP) does not foster learning and memory (Stent, 1973; Bienenstock et al., 1982). Indeed, Hebb had proposed that synaptic connections that were not utilized could weaken passively, however, several contradictions (including the ones mentioned above) indicated the necessity for an active depressive synaptic mechanism (Stent, 1973; Sejnowski, 1977; Bienenstock et al., 1982), thus paving the way for long-term depression (LTD) and bidirectional and STDP to be unraveled (Lynch et al., 1977; for review see Abbott and Nelson, 2000).

One pertinent question that arises based on these findings is in the context of cortical networks. What can be said about the topological network arrangement of cortical neurons? Through the past several years, sharp electrode and whole-cell recordings from multiple neurons have indicated that not only are the connection probabilities different for the multitude of neuron types, but also the numbers and distributions of synapses can vary widely (Feldmeyer et al., 1999, 2002; Reyes and Sakmann, 1999; Thomson et al., 2002; Kampa et al., 2006a; Frick et al., 2008). At the outset, these data provide ready evidence for synaptic clustering, however, these types of clustering might likely also arise from other mechanisms attributable to topographical, genetic, and neuron-specific connectivity rules (Callaway and Katz, 1993; Stepanyants et al., 2004; Luo et al., 2008; Chédotal and Richards, 2010).

Early evidence for synaptic clustering between neurons of the same type in the microcircuit came about with the discovery that TTL5 neurons are bidirectionally more frequently connected as opposed to their unidirectional connection probability (Markram et al., 1997a,b). A rigorous and methodical study of triplets of TTL5 neurons by Sen Song in Dmitri Chklovskii’s group (Song et al., 2005) demonstrated the existence of three-cell connectivity motifs in the neocortical microcircuit.

A recent study by Perin et al. demonstrated, by recording simultaneously from clusters of up to 12 TTL5 neurons, that both connection probability and synaptic strength can be predicted based on the number of common neighbors of a single TTL5 neuron (Perin et al., 2011). A rather simple clustering rule where connectivity is directly proportional to the number of common neighbors was found to account for “small world networks” of neurons, accurately predicting the connection probability between any two TTL5 neurons. The composite neurons of each group were found to be surprisingly distributed, typically more than 100 μm apart, allowing for multiple groups to be interlaced in the same space. This study led to the discovery of a synaptic organizing principle that groups neurons in a manner that is common across animals and hence, independent of individual experiences (Perin et al., 2011). The postnatal development of TTL5 neurons, however, is likely to be activity dependent and could involve mechanisms such as long-term microcircuit plasticity that has previously been identified at synaptic contacts underlying these connections (Le Bé and Markram, 2006).

### Synaptic Plasticity

Synapses are plastic. Governed by temporal patterns of pre- and postsynaptic activity, the process of synaptic plasticity is widely believed to underlie learning and memory. Postsynaptic activity is shaped by the active and passive properties of the dendritic arbor. Dendritic excitability regulates the plastic properties of the synapse over several time scales and stages of development (Desai et al., 1999a,b; Sjöström et al., 2001, 2008).

#### Plasticity in Local Microcircuits: The Ability to Rewire

Structurally, the local neocortical microcircuit is a *tabula rasa*, with each TTL5 axon forming several appositions with all neighboring TTL5 dendrites (Kalisman et al., 2005). Functionally, however, the constituent neurons of the microcircuit are fickle in choosing their postsynaptic partners, with synaptic contacts established only onto a small fraction of these targets. This blueprint equips the neocortical microcircuit with a high potential for plasticity enabling the formation of a multitude of functional microcircuits, which are continually transformed owing to the activity of constituent neurons.

Nir Kalisman and colleagues discovered that in the local neocortical microcircuit the *tabula rasa* configuration confers an all-to-all geometrical connectivity matrix between neighboring TTL5 neurons (Kalisman et al., 2005). This enables a reconfiguration of the microcircuit without any remodeling of arbors simply by the genesis or termination of synapses (boutons and spines) at existing physical appositions (Kalisman et al., 2005). This poses pertinent questions of whether the microcircuit is in a state of spontaneous preparedness to rapidly turn connections between TTL5 neurons on or off without any further regrowing of axons or dendrites?

Le Bé and Markram (2006) demonstrated a novel form of microcircuit plasticity where multisynaptic connections between TTL5 neurons were turned on or off over a time scale of several hours (Le Bé and Markram, 2006). New connections emerged as a result of bath application and periodic puffing of glutamate, while the disappearance of connections remained unaffected. These experiments provided direct evidence that excitation mediated by glutamate catalyzed the formation of new connections. The same study also provided insights on the strength of emerging and disappearing connections, in that emergent connections were found to be weaker than the existing ones. Interestingly, the synaptic connections that disappeared over a span of time were preferentially the weaker ones (with fewer synapses), underlying a co-operative mechanism that drives the formation and maintenance of multi-synapse connections in the neocortex (Fares and Stepanyants, 2009).

The phenomenon of microcircuit plasticity is likely triggered by new experiences, which removes the weakest connections and provides a grace period to test the merit of new emergent connections for their retention or elimination, endowing microcircuits with the ability to choose stronger and thus “fitter” connections in a Darwinian fashion (Le Bé and Markram, 2006). TTL5 synaptic connections are rarely if ever liaised by fewer than three contacts per connection. Microcircuit plasticity is thus an attractive contender for a candidate mechanism that selectively governs the number of putative synaptic contacts in TTL5 connections within the range of 4–8 contacts, as observed *in vitro*.

#### Redistribution of Synaptic Efficacy

bAPs trigger synaptic modifications as they temporally collide with or miss incoming EPSPs, treading the “delicate” line between Hebbian and anti-Hebbian synaptic modifications (Markram et al., 1997b; Bi and Poo, 1998; Sjöström et al., 2001, 2007, 2008; Kampa et al., 2006a, 2007; Letzkus et al., 2006; Sjöström and Häusser, 2006). The consequent change in synaptic strength from Hebbian pairing is not a uniform amplification of responses at all frequencies, but arises due to a redistribution of available synaptic efficacy.

Conventionally, synaptic plasticity has been evaluated by measuring the change in the amplitude of synaptic responses evoked by single-shock extracellular electrical stimulation of afferent fibers (Bliss and Lomo, 1973). The general behavior of a synapse cannot be conclusively extrapolated by unitary responses, therefore a train of presynaptic APs is essential to monitor gain changes at a synapse (Thomson et al., 1993; Markram and Tsodyks, 1996). A first demonstration of such synaptic gain changes, now referred to as redistribution of synaptic efficacy (RSE), showed that the absolute synaptic efficacy of TTL5 connections remained unaffected following high-frequency presynaptic stimulation (Markram and Tsodyks, 1996). It was also noted that the entire synaptic response to the high-frequency presynaptic AP train was not uniformly increased, but instead the existing synaptic efficacy was redistributed (Markram and Tsodyks, 1996). The same study also suggested likely mechanisms contributing to the increased use of the existing efficacy in TTL5 synapses, through either an increase in the probability of neurotransmitter release after Hebbian-pairing or by an increase in the affinity of postsynaptic glutamatergic receptors, provided the receptors are not completely saturated.

#### Excitability and Plasticity: Two Sides of the Same Coin

Although the morphology of the dendritic arbor remains mostly untransformed, its electrical properties can change in an activity-dependent manner over seconds to hours and perhaps even days, implying that dendritic learning rules exist in conjunction with synaptic rules (Sjöström et al., 2007; for review see Sjöström et al., 2008). Synapses convey information through the dendrites to the soma, triggering axonal APs as the final output. This process is symbiotic, where synaptic activity regulates dendritic excitability, and the dendritic arbor in turn exerts influence through the induction of synaptic plasticity.

A bAP invading the TTL5 dendritic arbor sparks synaptic modification (Markram et al., 1997b; Sjöström et al., 2001, 2007, 2008; Dan and Poo, 2004, 2006; Kampa and Stuart, 2006; Letzkus et al., 2006; Sjöström and Häusser, 2006; Kampa et al., 2007). Manipulating the relative timing of pre and postsynaptic APs led to the discovery of a watershed learning rule where the coincidence of postsynaptic APs and unitary EPSPs induced changes in EPSP amplitude in TTL5 neurons (Markram et al., 1997b). It was discovered that if the presynaptic AP preceded the postsynaptic AP by as little as 10 ms, the connection was strengthened, leading to LTP. On the contrary, if the postsynaptic AP preceded the presynaptic AP, the connection was weakened, causing LTD. The phenomenon of the regulation of synaptic efficacy by coincidental APs and EPSPs, christened STDP has established itself as an attractive model for learning at the level of single cells across several brain regions (Magee and Johnston, 1997; Markram et al., 1997b; Bi and Poo, 1998; Abbott and Nelson, 2000; Sjöström et al., 2001; Kampa et al., 2007; also see Lisman and Spruston, 2005). The change in synaptic strength plotted as a function of the relative timing of pre- and postsynaptic APs is referred to as the STDP function or learning window and varies between synapse types (Abbott and Nelson, 2000).

In classical STD, the timing of pre and postsynaptic APs induces LTP by depolarizing and unblocking NMDA receptors (Magee and Johnston, 1997; Markram et al., 1997b; Kampa et al., 2007). Under situations wherein bAPs completely fail to invade the dendritic arbor, STDP can still be induced if the bAPs are salvaged by sufficient postsynaptic depolarization. This can either be achieved by current injection during whole-cell recordings or by evoking bursts of APs (Sjöström et al., 2001; Letzkus et al., 2006; Sjöström and Häusser, 2006; Kampa et al., 2007). High-frequency bursts of APs occur naturally in TTL5 neurons and influence the initiation of dendritic spikes, consequently depolarizing the dendritic arbor (Lisman, 1997; Larkum et al., 1999a; Williams and Stuart, 1999; Kampa and Stuart, 2006). It has been demonstrated that only AP bursts above a critical firing frequency (~100 Hz) initiate dendritic spikes (Larkum et al., 1999a; Kampa and Stuart, 2006). Compelling evidence also indicates that AP bursts are required to exceed a critical frequency to bring about STDP, implying an imperative requirement of dendritic spikes (Kampa et al., 2006b). Similarly, the induction of STDP at synapses on basal and apical dendrites of TTL5 neurons can be blocked by the inhibition of dendritic spikes by voltage-gated Ca^2+^ channel antagonists (Kampa et al., 2006b; Letzkus et al., 2006). In summary, all these key findings strongly indicate that global dendritic spikes during AP burst firing are indispensable for the induction of LTP during low frequency pairing.

The question that arises then is how does dendritic synapse location impact the induction of STDP? Synapses onto different regions of the TTL5 dendritic arbor transmit a melange of information, which could be integrated in numerous ways. In concurrence with this notion, proximal synapses function by directly depolarizing the axo-somatic compartment, whereas information conveyed by distal synapses is mostly integrated through the initiation of regenerative dendritic spikes. Recent studies have focused on deciphering the relevance of dendritic synapse location for STDP induction in principal neocortical neurons. The first study by Rob Froemke et al. (2005) to address the location dependence of STDP indicated that the time window for LTD induction in layer 2/3 pyramidal neurons (L2/3PCs) is broader for inputs from distal synapses (Froemke et al., 2005). It was discovered that Ca^2+^ dependent suppression of NMDARs in the distal dendrites led to a broadening of the time window for LTD induction. Two other studies have investigated distance dependent STDP induction exclusively in TTL5 neurons. Jesper Sjöström in Michael Häusser’s laboratory demonstrated that pairing trains of APs and EPSPs led to LTP at proximal inputs (Sjöström and Häusser, 2006). In contrast, the same paradigm induced LTD at distally located inputs. Distal LTD was induced even while postsynaptic firing was absent, but could be transformed to LTP fostered by bAPs following sufficient dendritic current injection. An independent and complementary study by Letzkus et al. (2006) demonstrated that pairing unitary layer 2/3 inputs with bursts of APs at positive times led to LTP at proximal synapses and LTD at distal synapses in TTL5 neurons. On the contrary, negative pairings had the opposite effect, inducing LTD at proximal inputs and LTP of distal inputs in TTL5 neurons (Letzkus et al., 2006). Recent work by Gordon et al. demonstrated that TTL5 basal dendrites manifest compartments of plasticity (Gordon et al., 2006). While synapses onto proximal basal dendrites are modified when paired with the global activity of the neuron, in distal basal dendrites NMDA spikes serve as a local postsynaptic signal for induction of LTP.

#### Modulation of Intrinsic Excitability and Plasticity

Several studies have investigated the influence of neuromodulators such as dopamine (DA), and acetylcholine (ACh) on neuronal excitability in the juvenile rodent neocortex (Gulledge and Jaffe, 1998; Gulledge and Stuart, 2005). Allan Gulledge and David Jaffe measured the effect of DA on the membrane properties of TTL5 neurons and discovered that over a range of concentrations, DA decreased the excitability of TTL5 neurons (Gulledge and Jaffe, 1998). In another study, Gulledge and Jaffe demonstrated that the dopaminergic modulation of TTL5 neurons occurs through at least three different mechanisms: (a) DA was found to inhibit AP generation by enhancing spontaneous inhibitory synaptic input; (b) DA decreased the input resistance of TTL5 neurons; and (c) DA triggered a delayed and prolonged enhancement of excitability (Gulledge and Jaffe, 2001). Although DA inhibits AP generation in TTL5 neurons, it does not influence the back-propagation of APs, and the initiation of local dendritic spikes in these neurons (Gulledge and Stuart, 2003). These results indicate that DA likely does not play a modulatory role on the dendritic properties of TTL5 neurons, however this entails further investigation. However, recent evidence has emerged on the selective modulation of K+ currents at proximal unmyelinated segments of the TTL5 primary axon collateral (Yang et al., 2013). DA receptors, particularly D1 and D2 suppressed and enhanced K+ currents respectively, owing to differential regulation in the activity of cyclic AMP-dependent protein kinase signaling pathways (Yang et al., 2013). DA modulated the time course and kinetics of APs recorded directly from the TTL5 axon. DA could therefore play a substantial role in governing neuronal signaling through the active maintenance of axonal K^+^ currents.

Cholinergic release modulates TTL5 neurons in a variety of ways. ACh reduces the rate of synaptic depression between TTL5 neurons without affecting the so-called stationary EPSPs following presynaptic stimulation (Tsodyks and Markram, 1997). This suggests that ACh attenuates temporal coding in TTL5 neurons. Angel Nuñez et al. (2012) provided evidence that ACh enhances EPSCs through nicotinic and M1 muscarinic receptors while diminishing IPSCs through nicotinic and M2 muscarinic receptors. Consequently, the aggregate effects contributed to increased excitability, generation of Ca^2+^ spikes and bursts of APs when inputs exclusive to basal dendrites were stimulated (Nuñez et al., 2012). ACh therefore exerts influence in maintaining the excitation-inhibition balance by switching TTL5 neurons into a bursting regime causing response enhancement spelling profound implications for necortical function. Activity in the medial prefrontal cortex (mPFC) is critically implicated by ACh which critically modulates the PFC during behavioral tasks that require intense attention and focus. It has been demonstrated that prefrontal ACh release exists across multiple time-scales altering phasic dynamics as a result (Parikh et al., 2007). In particular, during coincident presynaptic and postsynaptic activity, nicotinic acetylcholine receptors (nAChRs) enhance GABAergic synaptic transmission, profoundly impacting dendritic calcium signals and AP propagation in TTL5 neurons (Couey et al., 2007). Rapid increase of ACh exhibits laminar specificity by selectively modulating PCs in layers 2/3 and 5 (Poorthuis et al., 2013). A medley of ionotropic nAChRs modulates the dynamics of TTL5 neurons in several ways, which includes raising the threshold for the induction of spike-timing dependent plasticity (Couey et al., 2007).

The exclusive dendritic domains of TTL5 neurons can also modulate synaptic plasticity. Gordon et al. (2006) discovered that pairing of APs and EPSPs led to LTP induction in proximal basal dendrites, however, in distal basal dendrites LTP could be induced only when synaptic activation paired with the local application of brain-derived neurotrophic factor (BDNF) initiated local NMDA spikes. Although BDNF is known to have a modulatory effect on cortical synaptic plasticity (Desai et al., 1999b), experiments by Gordon et al. (2006) could, for instance, provide a basis to differentiate between synapses occurring on proximal and distal parts of the TTL5 basal dendritic arbor, aided by BDNF application which serves as a “gating molecule”. Furthermore, cholinergic modulation can directly influence synaptic plasticity by shifting the polarity of plasticity, suggesting different modes for Hebbian modifications in TTL5 neurons (Stiefel et al., 2005).

In a series of recent experiments, Trevor Hamilton et al. demonstrated that neuropolypeptide Y (NPY) modulates the induction of LTD by suppressing Ca^2+^ influx induced by trains of bAPs (Hamilton et al., 2013). In what appears as a precise design strategy, release of NPY specifically targets the distal TTL5 dendrite occurring in the superficial layers. NPY receptors localized on distal dendritic processes selectively modulate LTD by regulating Ca^2+^ influx and regenerative potentials in TTL5 apical dendrites and tufts.

The influence of local TTL5 excitability on global network activity can be significantly altered by the use of anesthetics. *In vivo* experiments measuring TTL5 excitability have thus far been exclusively carried out under the use of anesthetics, particularly urethane, barbiturate, or ketamine, which can profoundly impact the active properties of TTL5 dendrites and intrinsic firing properties (Svoboda et al., 1997; Helmchen et al., 1999; Larkum and Zhu, 2002). It is therefore imperative that the consequences of anesthetic modulation of TTL5 excitability are well understood prior to deriving conclusions from *in vivo* measurements. A comprehensive study by Sarah Potez in Matthew Larkum’s group identified the impact of anesthetics on TTL5 excitability both *in vitro* and *in vivo* (Potez and Larkum, 2008). In summary, while local Ca^2+^ dendritic spikes were not totally abolished by any of the commonly used anesthetics, urethane depolarized the firing threshold causing hyper-excitability; dose-response characteristic of barbiturate inhibited spontaneous spiking activity; ketamine caused hyper-excitability with a broadening of dendritic Ca^2+^ spikes and lowered the critical frequency for their induction (Potez and Larkum, 2008). Although the use of anesthetics does not impair the active propagation of dendritic spikes *in vivo*, their direct modulation of the kinetics and distribution of ion channels in TTL5 dendrites warrants further investigation.

### Pathophysiology and Disease

Over the years, a wealth of information has accumulated on TTL5 function, what then is our current understanding on TTL5 dysfunction? TTL5 dysfunction has been strongly implicated to a host of maladies such as schizophrenia, epilepsy, autism, anxiety and depression critically disrupting cortical activity (Black et al., 2004; Traub et al., 2005; Lytton, 2008).

Schizophrenia is a neuropsychiatric disorder marked by pronounced deterioration of thinking and emotional responsiveness. The basal dendritic arbor in prefrontal cortical TTL5 neurons atrophies in size due to schizophrenia, and the consequent abnormal dendritic outgrowth leads to a reduction in cortical neuropil, thereby causing a decrease in connectivity between TTL5 neurons (Black et al., 2004). The reduced prefrontal neuropil could also be associated with less dopaminergic innervation of the deep layers of the prefrontal cortex, owing to schizophrenia (Garey et al., 1998; Black et al., 2004).

Epilepsy is one of several episodic disorders of the brain, characterized by recurrent synchronous neuronal activity. Epilepsy is not necessarily a single disorder, but manifests itself in several forms, including multiple sclerosis, transient ischemic attacks and migraine, all dynamical disorders that become apparent over time (Khosravani and Zamponi, 2006; Lytton, 2008). Recent evidence indicates that distinct forms of epilepsy are linked to changes in the efficacy of the I_h_ current carried by HCN channels (Di Pasquale et al., 1997; for review see Santoro and Baram, 2003).

The deficit in I_h_ mediated functions may contribute to the development and onset of spontaneously occurring hyper-excitability in neocortical pyramidal neurons in a rat model of absence seizures (Strauss et al., 2004). Furthermore, in a genetic rat model of absence epilepsy, Maarten Kole in Greg Stuart’s group showed that a loss of dendritic I_h_ recruits Ca^2+^ channels to amplify bAP triggered dendritic Ca^2+^ spikes leading to an increase in burst firing (Kole et al., 2007a). Therefore, the deficit of dendritic HCN channels in TTL5 neurons provides a somato-dendritic mechanism for increasing the synchronization of cortical output, and is therefore likely to play an important role in the generation of absence seizures (Kole et al., 2007a). Additionally, recent experiments have shown that sensory deprivation in neocortex increases the intrinsic excitability of TTL5 neurons through epileptic seizures from increased dendritic Ca^2+^ arising through a deficit of HCN expression (Breton and Stuart, 2009). These preliminary findings have contributed to a better understanding of the cortical basis of idiopathic generalized epilepsies and bolster the idea that the mechanisms involved in HCN expression hold promise as therapeutic targets for the treatment of absence seizures.

The K^+^/Cl^−^ cotransporter (KCC2) is crucial in maintaining low [Cl^−^]_i_ resulting in hyperpolarizing GABA responses. A decrease in KCC2 after neuronal injuries resulted in increases in [Cl^−^]_i_ and enhanced neuronal excitability in TTL5 neurons due to depolarizing GABA responses (Jin et al., 2006). Perforated-patch recordings revealed the functional consequence of KCC2 down-regulation in chronically injured neocortex indicating that a positive shift in the GABAergic reversal potential due to Cl^−^ extrusion (directly attributed to KCC2 down-regulation) caused acute epileptogenesis in pathophysiological TTL5 neurons (Jin et al., 2006).

Autism is a developmental disorder of neurological origin, primarily affecting social cognition. The etiology of autism has not been conclusively established yet, but genetic and environmental alterations are believed to confer vulnerability to this neurological disorder (Rubenstein and Merzenich, 2003). Recent studies have focussed on animal models of autism to explore changes in molecular, synaptic and cellular properties in pathological TTL5 neurons (Rinaldi et al., 2007a,b).

Rinaldi et al. investigated the postnatal effects of embryonic exposure to valproic acid (VPA) on TTL5 neurons of juvenile rat somatosensory cortex through whole cell patch-clamp recordings, and discovered that a single prenatal injection of VPA caused a significant enhancement of the local recurrent connectivity formed by TTL5 neurons (Rinaldi et al., 2007b). The connections between these pathological TTL5 neurons led to weaker synaptic responses, and their intrinsic excitability was also weakened. Furthermore, the mean number of potential synaptic contacts diminished (from an average of ~5.5 in control neurons to ~3.3 in pathological neurons) following exposure to VPA (Rinaldi et al., 2007b). In a complementary study, Rinaldi et al. (2007a) demonstrated that a single prenatal injection of VPA caused a surprisingly selective enhancement of NMDAR subunits NR2A and NR2B (Rinaldi et al., 2007a). This selective enhancement translated into enhanced NMDAR mediated synaptic currents and a marked amplification of synaptic plasticity through LTP in TTL5 neurons. These results provide preliminary insight on some of the potential core symptoms observed in humans prenatally exposed to VPA, and hold promise for the therapeutic treatment of autism.

### Five Decades of TTL5 Research: What Lies Ahead?

We have endeavored to provide a unifying view of the dendritic, axonal, synaptic properties and plasticity of TTL5 neurons, and to a lesser extent the pathophysiology of TTL5 neurons from more than five decades of research. In this review, a degree of inevitable recurrence exists in the description of some key phenomena. A high level of interdependence is evident between dendritic excitability and synaptic plasticity and vice-versa, where on the one hand activity-dependent regulation of dendritic excitability induces synaptic plasticity, and synaptic plasticity controls dendritic computations on the other.

Although we have learnt much more about the function of TTL5 neurons in the past several decades than ever before, this is still the tip of the iceberg. To reveal all anatomical and physiological aspects seems still far in the future. With the advent of newer *in vivo*, *in vitro* and *in silico* approaches for targeted experiments on TTL5 neurons, specific questions can be asked about their occurrence and generalization across different species and/or cortical regions, robustness and redundancy in local circuit organization, gene expression changes across developmental time scales and activity patterns, protein transport and localization across different morphological regions, gene and protein networks underlying specific functions, the biophysical role of spines, possible cell assemblies based on afferent input and efferent output, the neuromodulation of plasticity, how different parts of the dendrite participate in the emergence of receptive fields, integration of translaminar, transcolumnar and transregional synaptic input, to outline a few. Gaining a grasp of a how single TTL5 neuron is actively modulated by the myriad of excitatory and inhibitory synapses impinging onto different dendritic domains is fundamental to better understand the multimodal information processing capabilities of these neurons. The pursuit to unravel the anatomy and physiology of TTL5 neurons will increase our understanding of their pivotal roles in neuronal microcircuitry and why our neuronal network is so wired to make us who we are.

## Conflict of Interest Statement

The authors declare that the research was conducted in the absence of any commercial or financial relationships that could be construed as a potential conflict of interest.

## Abbreviations

ACh: acetylcholine
AIS: axon initial segment
AMPAR: AMPA receptor
AMP: adenosine monophosphate
AP: action potential
bAP: back-propagating action potential
bAC: back-propagation activated Ca^2+^ spike
BDNF: brain-derived neurotrophic factor
BK: big-conductance Ca^2+^ dependent K^+^ channel
CV: coefficient of variation
DA: dopamine
HCN: hyperpolarization-activated cation
IR-DIC: infrared differential interference contrast
KAR: kainate receptor
KCC2: K^+^/Cl^−^ cotransporter
L2/3PC: layer 2/3 pyramidal neuron
TTL5: thick-tufted layer 5
NMDAR: NMDA receptor
P: postnatal day
PSD: postsynaptic density
RSE: redistribution of synaptic efficacy
SK: small-conductance Ca^2+^ dependent K^+^ channel
STDP: spike-timing dependent synaptic plasticity
VPA: valproic acid.
